# Chromosomal-level assembly of yellow catfish genome using third-generation DNA sequencing and Hi-C analysis

**DOI:** 10.1093/gigascience/giy120

**Published:** 2018-09-20

**Authors:** Gaorui Gong, Cheng Dan, Shijun Xiao, Wenjie Guo, Peipei Huang, Yang Xiong, Junjie Wu, Yan He, Jicheng Zhang, Xiaohui Li, Nansheng Chen, Jian-Fang Gui, Jie Mei

**Affiliations:** 1 College of Fisheries, Key Laboratory of Freshwater Animal Breeding, Ministry of Agriculture, Huazhong Agricultural University, Wuhan, Hubei, 430070, China; 2 Wuhan Frasergen Bioinformatics, East Lake High-Tech Zone, Wuhan, Hubei, 430075, China; 3 State Key Laboratory of Freshwater Ecology and Biotechnology, Institute of Hydrobiology, Chinese Academyof Sciences, University of the Chinese Academy of Sciences, Wuhan, Hubei, 430072, China; 4 Oceanology, Chinese Academy of Sciences, Qingdao, Shandong, 266071, China; 5 Department of Molecular Biology and Biochemistry, Simon Fraser University, Burnaby, Canada

**Keywords:** yellow catfish, PacBio, Hi-C, genomics, chromosomal assembly

## Abstract

**Background:**

The yellow catfish, *Pelteobagrus fulvidraco*, belonging to the Siluriformes order, is an economically important freshwater aquaculture fish species in Asia, especially in Southern China. The aquaculture industry has recently been facing tremendous challenges in germplasm degeneration and poor disease resistance. As the yellow catfish exhibits notable sex dimorphism in growth, with adult males about two- to three-fold bigger than females, the way in which the aquaculture industry takes advantage of such sex dimorphism is another challenge. To address these issues, a high-quality reference genome of the yellow catfish would be a very useful resource.

**Findings:**

To construct a high-quality reference genome for the yellow catfish, we generated 51.2 Gb short reads and 38.9 Gb long reads using Illumina and Pacific Biosciences (PacBio) sequencing platforms, respectively. The sequencing data were assembled into a 732.8 Mb genome assembly with a contig N50 length of 1.1 Mb. Additionally, we applied Hi-C technology to identify contacts among contigs, which were then used to assemble contigs into scaffolds, resulting in a genome assembly with 26 chromosomes and a scaffold N50 length of 25.8 Mb. Using 24,552 protein-coding genes annotated in the yellow catfish genome, the phylogenetic relationships of the yellow catfish with other teleosts showed that yellow catfish separated from the common ancestor of channel catfish ∼81.9 million years ago. We identified 1,717 gene families to be expanded in the yellow catfish, and those gene families are mainly enriched in the immune system, signal transduction, glycosphingolipid biosynthesis, and fatty acid biosynthesis.

**Conclusions:**

Taking advantage of Illumina, PacBio, and Hi-C technologies, we constructed the first high-quality chromosome-level genome assembly for the yellow catfish *P. fulvidraco*. The genomic resources generated in this work not only offer a valuable reference genome for functional genomics studies of yellow catfish to decipher the economic traits and sex determination but also provide important chromosome information for genome comparisons in the wider evolutionary research community.

## Data Description

### Introduction

**Figure 1:**
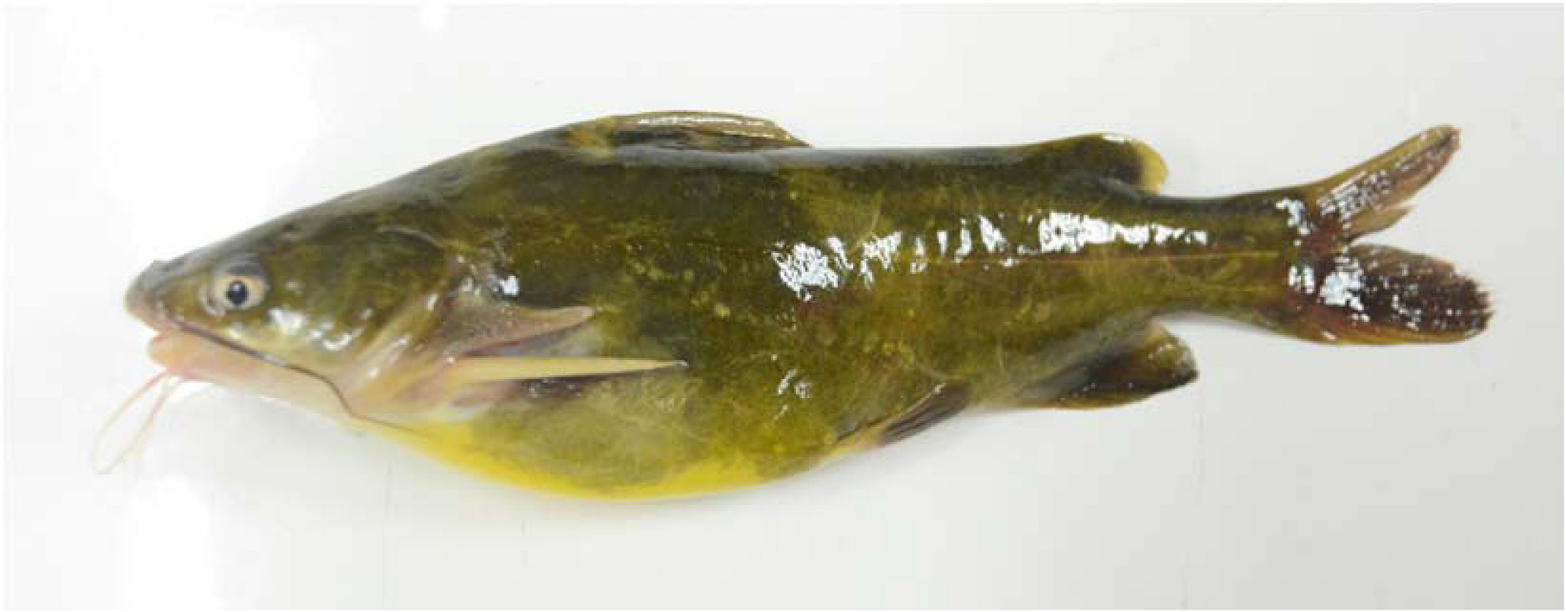
A yellow catfish, *Pelteobagrus fulvidraco*. The fish was collected from the breeding center of Huazhong Agricultural University in Wuhan City, Hubei Province, China.

The yellow catfish, *Pelteobagrus fulvidraco* (Richardson, 1846; National Center for Biotechnology Information [NCBI] Taxonomy ID:  1 234 273; Fishbase ID: 28 052) is a teleost fish belonging to the order Siluriformes (Fig. 1) and is an economically important freshwater fish species in Asia [1]. In recent years, the yellow catfish has become one of the most important aquaculture species in China with an increasing market value because of9 its high meat quality and lack of intermuscular bones next to the spine [2]. However, due to ultra-intensive aquaculture and loss of genetic diversity, artificial breeding of yellow catfish is facing tremendous challenges such as germplasm degeneration and poor disease resistance [3]. Meanwhile, as an XY sex-determining type fish species, yellow catfish is also an excellent model for studying sex determination and sexual size dimorphism in fish [4, 5]. As female and male yellow catfish exhibit remarkable sex dimorphism in their growth rate, with adult yellow catfish males about two- to three-fold bigger than the females. In the last decade, sex-specific allele markers were developed and YY super-male fish were generated from gynogenesis of XY physiological female fish. Finally, XX male, XY female, YY super-male, and females have been created and provide a unique model to study sex determination in fish species [1, 6, 7]. Recently, transgene and gene knockout technologies have been successively applied in yellow catfish to reveal the function of the *domain present in PSD-95, Dlg and ZO-1/2 (pfpdz1*) gene, a novel PDZ domain-containing gene in whose intron the sex-linked marker was located. The *pfpdz1* gene plays an important role in male sex differentiation and maintenance in yellow catfish [8]. Taken together, these features provide a platform for gene-editing methods to study gene function.

In spite of the importance of yellow catfish both in sex-determination research and in aquaculture, the genomic resources for the species are still limited. To date, only transcriptome, simple sequence repeat, and single-nucleotide polymorphism (SNP) data have been reported for yellow catfish [5] and the genome sequence for this important species is still missing, hindering the identification of genome-based functional genes identification controlling important economic traits and the application of genome-assisted breeding in yellow catfish. In this work, we combined genomic sequencing data from Illumina short reads and Pacific Bioscience (PacBio) long reads to generate the first reference genome for yellow catfish. Also, we applied Hi-C data to scaffold the genome sequences to the chromosomal level. The completeness and continuity of the genome were comparable with that of other model teleost species. We believe that the high-quality reference genome generated in this work will definitely facilitate research on population genetics and functional gene identification related to important economic traits and the sex determinant for yellow catfish, which will in turn accelerate the development of more efficient sex-control techniques and improve the artificial breeding industry for this economically important fish species.

### Sample and sequencing

A XX genotype female yellow catfish (Fig. 1), reared in the breeding center of Huazhong Agricultural University in Wuhan City, Hubei Province, was used for preparing DNA for sequencing. To obtain sufficient high-quality DNA molecules for the PacBio Sequel platform (Pacific Biosciences , Menlo Park, CA, USA), one yellow catfish was dissected and fresh muscle tissues were used for DNA extraction using the phenol/chloroform extraction method as in a previous study [9]. The quality of the DNA was checked by agarose gel electrophoresis, and excellent integrity DNA molecules were observed. Other tissues, including ocular, skin, muscle, gonadal, intestinal, liver, kidney, blood, gallbladder, and air bladder,were snap-frozen in liquid nitrogen for at least 1 hour and then stored at −80°C.

The extracted DNA molecules were sequenced with both Illumina HiSeq X Ten platform (Illumina Inc., San Diego, CA, USA) and PacBio Sequel platforms. Short reads generated from the Illumina platform were used for the estimation of the genome size, the level of heterozygosity, and repeat content of the genome, and long reads from the PacBio platform were used for genome assembly. To this end, one library with an insertion length of 250 bp was generated for the HiSeq X Ten platform and three 20-kb libraries were constructed for the PacBio platform according to the manufacturers' protocols, resulting in the generation of ∼51.2 Gb short reads and ∼38.9 Gb long reads, respectively (Table 1). The polymerase and subreads N50 length reached 21.3 kb and 16.2 kb, providing ultra-long genomic sequences for the following assembly.

**Table 1:** Sequencing data generated for yellow catfish genome assembly and annotation

Library type	Platform	Library size (bp)	Data size (Gb)	Application
Short reads	HiSeq X Ten	250	51.2	Genome survey and genomic base correction
Long reads	PacBio SEQUEL	20,000	38.9	Genome assembly
Hi-C	HiSeq X Ten	250	146.1	Chromosome construction

Note that paired-end 150 bp reads were generated from the Illumina HiSeq X Ten platform.

### Genome features estimation from *k*-mer method

The short reads from the Illumina platform were quality filtered by HTQC v1.92.3 [10] using the following method. First, the adaptors were removed from the sequencing reads. Second, read pairs were excluded if any one end had an average quality lower than 20. Third, ends of reads were trimmed if the average quality was lower than 20 in the sliding window size of 5 bp. Finally, read pairs with any end shorter than 75 bp were removed.

The quality-filtered reads were used for genome size estimation. Using the *k*-mer method described in a previous method [11], we calculated and plotted the 17-mer depth distribution in Supplementary Fig. S1. The formula G = N_17-mer_/D_17-mer_, where the N_17-mer_ is the total number of 17-mers and D_17-mer_ denotes the peak frequency of 17-mers, was used to estimate the genome size of yellow catfish. We estimated a genome size of 714 Mb, as well as a heterozygosity rate of 0.45% and repeat ratio of 43.31%. To confirm the robustness of the genome size estimation, we performed additional analysis with *k*-mer of 21, 25, and 27 and found the estimated genome size ranged from 706 to 718 Mb (Supplementary Table S1).

### Genome assembly by third-generation long reads

With six single-molecular real-time cells in the PacBio Sequel platform, we generated 38.9 Gb subreads by removing adaptor sequences within sequences. The mean and N50 length were 9.8 and 16.2 kb, respectively. The long subreads were used for genomic assembly of yellow catfish. First, the Falcon v0.3.0 package [12] with a parameter of length_cutoff as 10 kb and pr_length_cutoff as 8 kb was used. As a result, we obtained a 690-Mb genome with a contig N50 length of 193.1 kb. Second, canu v1.5 [13] was employed separately for genome assembly with default parameters, leading to 688.6 Mb yellow catfish genome with contig N50 of 427.3 kb.

Although the size of the genome assembly from both Falcon and canu was comparable with the estimation based on the *k*-mer method, the continuity of the genome needed further improvement. Genome puzzle master (GPM) [14] is a tool to guide the genome assembly from fragmented sequences using overlap information among contigs from genomes [14]. Based on the complementarity of the two genomes, the contig could be merged and the gaps filled by sequences bridging the two contigs [15]. Taking advantage of the sequence complementation of the two assemblies from Falcon and canu, we therefore applied GPM [14] to merge long contigs using reliable overlaps between sequences. Finally, a ∼730-Mb genome assembly of yellow catfish with 3,564 contigs and contig N50/L50 of 1.1 Mb/126 was constructed. The final genome sequences were then polished by arrow [16] using PacBio long reads and by pilon release 1.12 [17] using Illumina short reads to correct errors in the base level. The length distribution for contigs in the final assembly is presented in Supplementary Fig. S2.

### *In situ* Hi-C library construction and chromosome assembly using Hi-C data

Hi-C is a technique that makes it possible to unbiasly identify chromatin interactions across the entire genome [18]. The technique was introduced in a genome-wide version of 3C (capturing chromosome conformation) [19] and was used as a powerful tool in the chromosome genome assembly of many projects in recent years [20]. In this work, Hi-C experiments and data analysis on blood samples were used for the chromosome assembly of the yellow catfish. Blood samples from the same yellow catfish used for genomic DNA sequencing was used for library construction for Hi-C analysis. We collected 0.1 mL blood that was cross-linked for 10 minutes with 1% final concentration fresh formaldehyde and quenched with 0.2 M final concentration glycine for 5 minutes. The cross-linked cells were subsequently lysed in lysis buffer (10 mMTris-HCl (pH 8.0), 10 mM NaCl, 0.2% NP40, and complete protease inhibitors [Roche]). The extracted nuclei were resuspended with 150 μL 0.1% sodium dodecyl sulfate (SDS) and incubated at 65°C for 10 minutes. Then SDS molecules were quenched by adding 120 μL water and 30 μL 10% Triton X-100 and incubated at 37°C for 15 minutes. The DNA in the nuclei was digested by adding 30 μL 10x New England Biolabs (NEB) buffer 2.1 (50 mM NaCl, 10 mM Tris-HCl, 10 mM MgCl_2_, 100 μg/mL bovine serum albumin (BSA), pH 7.9) and 150 U of MboI and incubated at 37°C overnight. On the next day, the MboI enzyme was inactivated at 65°C for 20 minutes. Next, the cohesive ends were filled in by adding 1 μL 10 mM dTTP, 1 μL 10 mM dATP, 1 μL 10 mM dGTP, 2 μL 5 mM biotin-14-dCTP, 14 μL water, and 4 μL (40 U) Klenow and incubated at 37°C for 2 hours. Subsequently, 663 μL water,120 μL 10x blunt-end ligation buffer (300 mM Tris-HCl, 100 mM MgCl_2_, 100 mM DTT, 1 mM ATP, pH 7.8), 100 μL 10% Triton X-100, and 20 U T4 DNA ligase were added to start proximity ligation. The ligation reaction was heldat 16°C for 4 hour. After ligation, the cross-linking was reversed with 200 µg/mL proteinase K (Thermo) at 65°C overnight. Subsequent chromatin DNA manipulations were performed using a method similar to the one described in the previous study [19]. DNA purification was achieved using QIAamp DNA Mini Kits (Qiagen) according to the manufacturer's instructions. Purified DNA was sheared to a length of ∼400 bp. Point ligation junctions were pulled down by Dynabeads MyOne Streptavidin C1 (Thermofisher) according to the manufacturer's instructions. The Hi-C library for Illumina sequencing was prepared using the NEBNext Ultra II DNA library Prep Kit for Illumina (NEB) according to the manufacturer's instructions. The final library was sequenced on the Illumina HiSeq X Ten platform (San Diego, CA, USA) with 150 paired-end mode.

**Figure 2:**
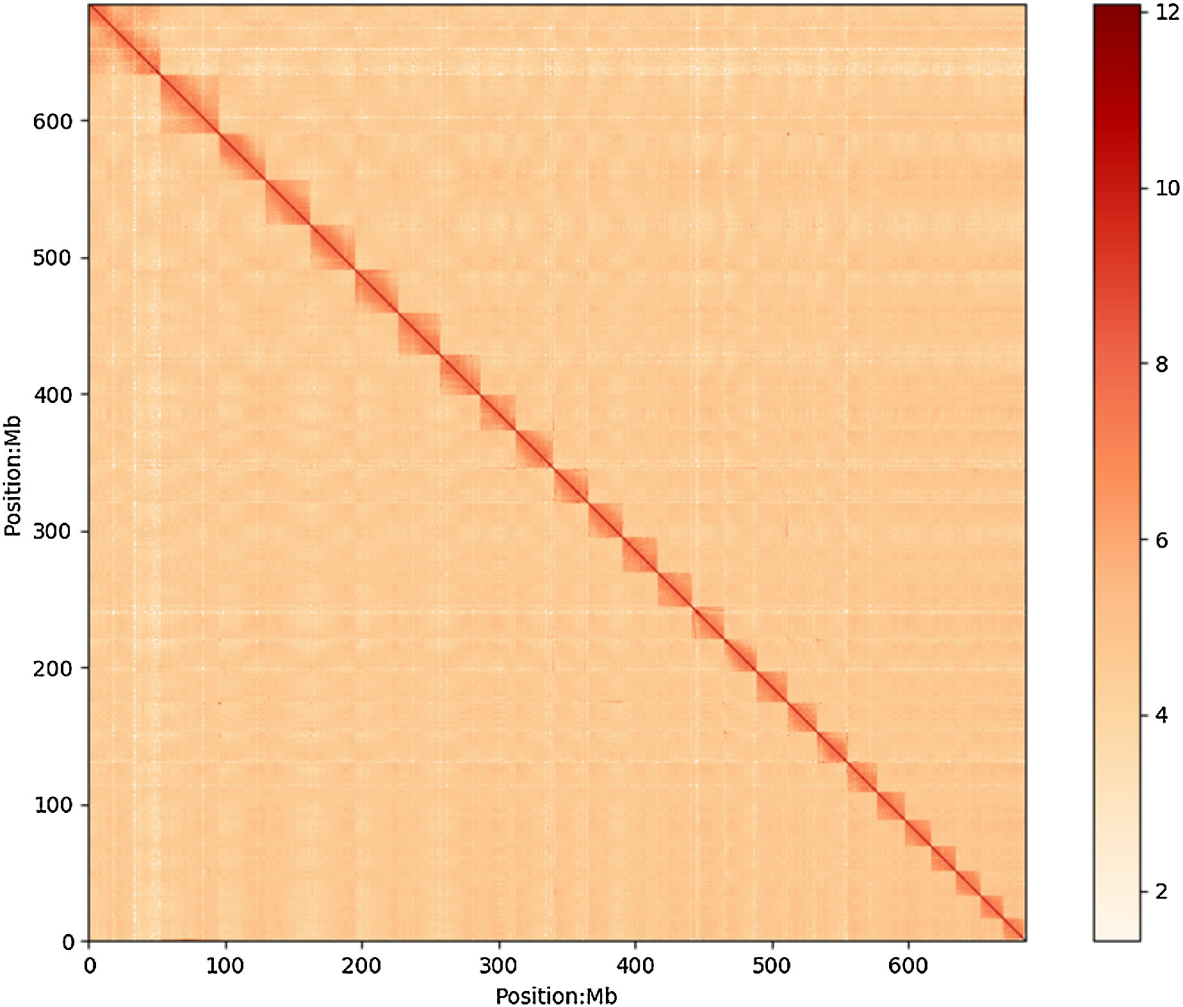
Yellow catfish genome contig contact matrix using Hi-C data. The color bar illuminates the logarithm of the contact density from red (high) to white (low) in the plot. Note that only sequences anchored on chromosomes are shown in the plot.

A total of 487 million raw reads were generated from the Hi-C library and were mapped to the polished yellow catfish genome using Bowtie 1.2.2 (RRID:SCR_005476) [21] with the default parameters. The iterative method was used to increase the interactive Hi-C reads ratio [22]. Two ends of paired reads were mapped to the genome independently, but only the reads for the two pairs that were uniquely mapped to the genome were used. Self-ligation, nonligation, and other invalid reads, such as StartNearRsite, PCR amplification, random break, LargeSmallFragments, and ExtremeFragments, were filtered using the method and hiclib as described in previous reports [23]. The contact count among each contig was calculated and normalized by the restriction sites in sequences (Fig. 2). We then successfully clustered 2,965 contigs into 26 groups with the agglomerative hierarchical clustering method in Lachesis [24], which was consistent with the previous karyotype analyses of *Pseudobagrus fulvidraco* [25]. Lachesis was further applied to order and orient the clustered contigs, and 2,440 contigs were reliably anchored on chromosomes, presenting 66.8% and 94.2% of the total genome by contig number and base count, respectively. Then, we applied juicebox [26] to correct the contig orientation and to remove suspicious fragments in contig to unanchored groups by visual inspection. Finally, we obtained the first chromosomal-level high-quality yellow catfish assembly with a contig N50 of 1.1 Mb and scaffold N50 of 25.8 Mb, providing a solid genomic resource for the following population and functional analysis (Table 2). We compared length distribution of contigs anchored and unanchored on chromosomes (Supplementary Fig. S3) and found that anchored contigs were significantly longer than unanchored contigs. We therefore speculated that short lengths of unanchored contigs limited effective Hi-C reads mapping, leading to insufficient supporting evidence for their clustering, ordering, and orientation on chromosomes. The gap distribution on chromosomes is shown in Supplementary Fig. S4. We found that gaps were mainly distributed at two ends of chromosomes, which could be explained by the repeat distribution at chromosome terminals. The length and the statistics of contigs and gaps of each chromosome are summarized in Supplementary Table S2.

**Table 2:** Statistics for genome assembly of yellow catfish

Sample ID	Length		Number	
	Contig** (bp)	Scaffold (bp)	Contig**	Scaffold
Total	731,603,425	732,815,925	3,652	1,227
Max	11,531,338	55,095,979	-	-
N50	1,111,198	25,785,924	126	11
N60	643,552	24,806,204	212	14
N70	333,994	22,397,207	373	17
N80	128,419	21,591,549	742	21
N90	59,682	16,750,011	1,634	25

Note that contigs were analyzed after the scaffolding based on Hi-C data.

**refers to contig sequences after removing gaps in the final genome assembly

### Genome quality evaluation

**Figure 3:**
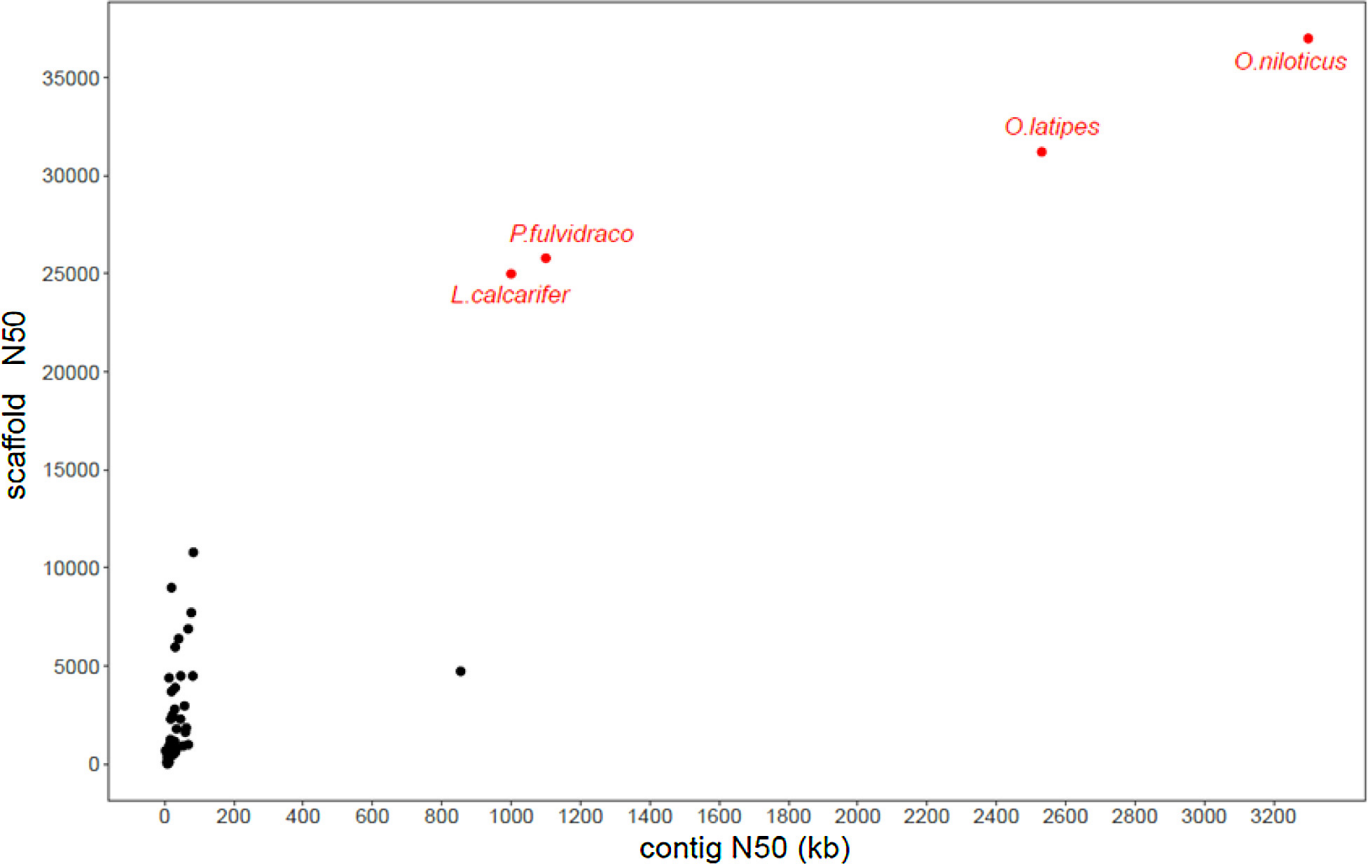
Genome assembly comparison of yellow catfish with other public teleost genomes. The *x*- and *y*-axis represent the contig and scaffold N50s, respectively. The genomes sequenced with third-generation sequencing are highlighted in red.

First, we compared the genome assembly continuity of the yellow catfish genome to those of other teleost species. We found that both contig and scaffold N50 lengths of the yellow catfish reached considerable continuity (Fig. 3), providing a high-quality genome sequencesfor the following functional investigations. The assembled genome was also subjected to Benchmarking Universal Single-Copy Orthologs (BUSCO) v3.0 [27] (RRID:SCR_015008, version 3.0) with the actinopterygii_odb9 database to evaluate the completeness of the genome. Among 4,584 total BUSCO groups searched, 4,179 and 92 BUSCO core genes were completed and partially identified, respectively, leading to a total of 91.2% BUSCO genes in the yellow catfish genome. After aligning short reads from the Illumina platform to the genome, the insertion length distribution for the sequencing library of 250 bp exhibited a single peak around the sequencing library length design (Supplementary Fig. S5). Paired-end reads data were not used during the contig assembly, thus the high alignment ratio and single peak insertion length distribution demonstrated the high quality of contig assembly for yellow catfish. Applying the Illumina short-read alignment to the reference genome of the yellow catfish by BWA 0.7.16 software (RRID:SCR_010910), we identified 21,143 homozygous SNP loci using the GATK (RRID:SCR_001876) package [28].

### Repeat and gene annotation

We first used Tandem Repeat Finder [29] to identify repetitive elements in the yellow catfish genome. RepeatModeler ([30], RRID:SCR_015027) was used to detect transposable elements (TEs) in the genome by a *de novo* manner. The *de novo* and known repeats library from Repbase [31] were then combined, and the TEs were detected by mapping sequences to the combined library in the yellow catfish genome using the software RepeatMasker 4.0.7 (RRID:SCR_012954) [32].

**Figure 4:**
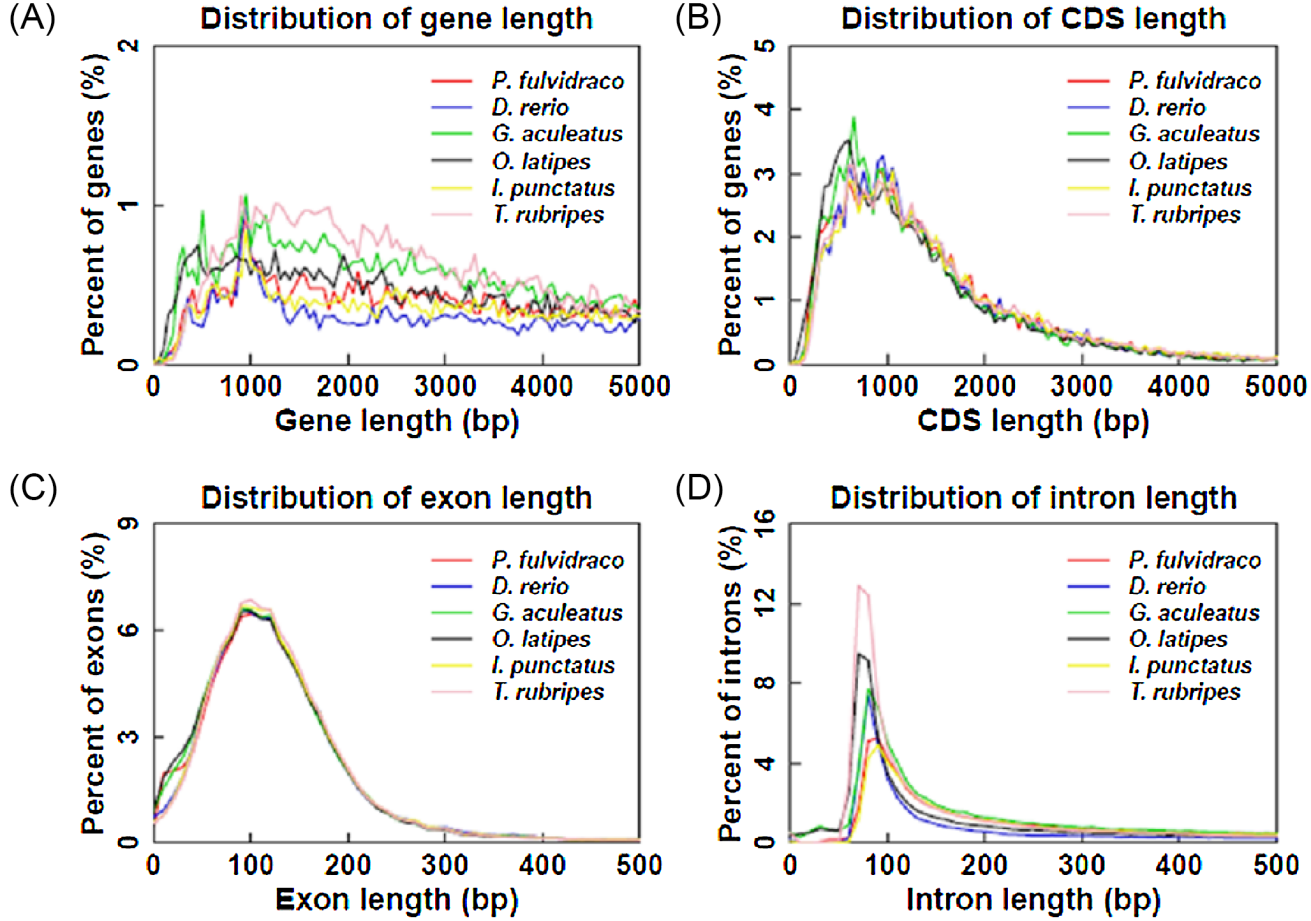
Length distribution comparison on total gene, CDS, exon, and intron of annotated gene models of the yellow catfish with other closely related teleost fish species. Length distribution of total gene (A), CDS (B), exon (C) ,and intron (D) were compared to those of *P. fulvidraco, D. rerio, G. aculeatus, O. latipes, I. punctatus*, and *T. rubripes*.

For protein-coding gene annotation, *de novo*-, homology-, and RNA-sequencing-based methods were used. Augustus (RRID:SCR_008417) [33] was used to predict coding genes in *de novo* prediction. For the homology-based method, protein sequences of closely related fish species, including *Astyanax mexicanus*, *Danio rerio*, *Gadus morhua*, *Ictalurus punctatus, Oryzias latipes, Takifugu rubripes, Tetraodon nigroviridis*, and *Oreochromis niloticus*, were downloaded from Ensembl [34] and aligned against to the yellow catfish genome using TBLASTN (RRID:SCR_011822) software [35]. Short reads from RNA-seq (SRR1845493) were also mapped on the genome using TopHat v2.1.1 (RRID:SCR_013035) [36], and the gene structure was formed using Cufflinks (RRID:SCR_014597) [37]. Finally, 24,552 consensus protein-coding genes were predicted in the yellow catfish genome by integrating all gene models by MAKER [38]. The gene number, gene length distribution, coding DNA sequence (CDS) length distribution, exon length distribution, and intron length distribution were comparable with those in other teleost fish species (Fig. 4).

Local Basic Local Alignment Search Tool (BLAST) X (RRID:SCR_001653) and BLASTN (RRID:SCR_001598) programs were used to search all predicted gene sequences to NCBI nonredundant protein (nr), no-redundant nucleotide (nt) Swissprot database with a maximal e-value of 1e^−5^ [39]. Gene ontology (GO) [40] and Kyoto Encyclopedia of Genes and Genomes (KEGG) [41] pathway annotations were also assigned to genes using the software Blast2GO [42]. As a result, 24,552 genes were annotated to at least one database (Table 3).

**Table 3:** Statistics for genome annotation of yellow catfish

Database	Number	Percent
InterPro	20,178	82.18
GO	14,936	60.83
KEGG ALL	24,025	97.85
KEGG KO	13,951	56.82
Swissprot	20,875	85.02
TrEMBL	24,093	98.13
NR	24,308	99.01
Total	24,552	

Note that the e-value threshold of the 1e-5 was applied during the homolog searching for the functional annotation.

### Gene family identification and phylogenetic analysis of yellow catfish

To cluster families from protein-coding genes, proteins from the longest transcripts of each gene from yellow catfish and other fish species, including *Ictalurus punctatus*, *Clupeaharengus*, *Danio rerio*, *Takifugu rubripes*, *Hippocampus comes*, *Cynoglossus semilaevis*, *Oryzias latipes*, *Gadus morhua*, *Lepisosteus oculatus*, *Dicentrarchus labrax*, and *Gasterosteus aculeatus*, were extracted and aligned to each other using BLASTP (RRID:SCR_001010) programs [39] with a maximum e-value of 1e^−5^. OrthMCL [43] was used to cluster gene family using protein BLAST results. As a result, 19,846 gene families were constructed for fish species in this work and 3,088 families were identified as single-copy ortholog gene families.

**Figure 5:**
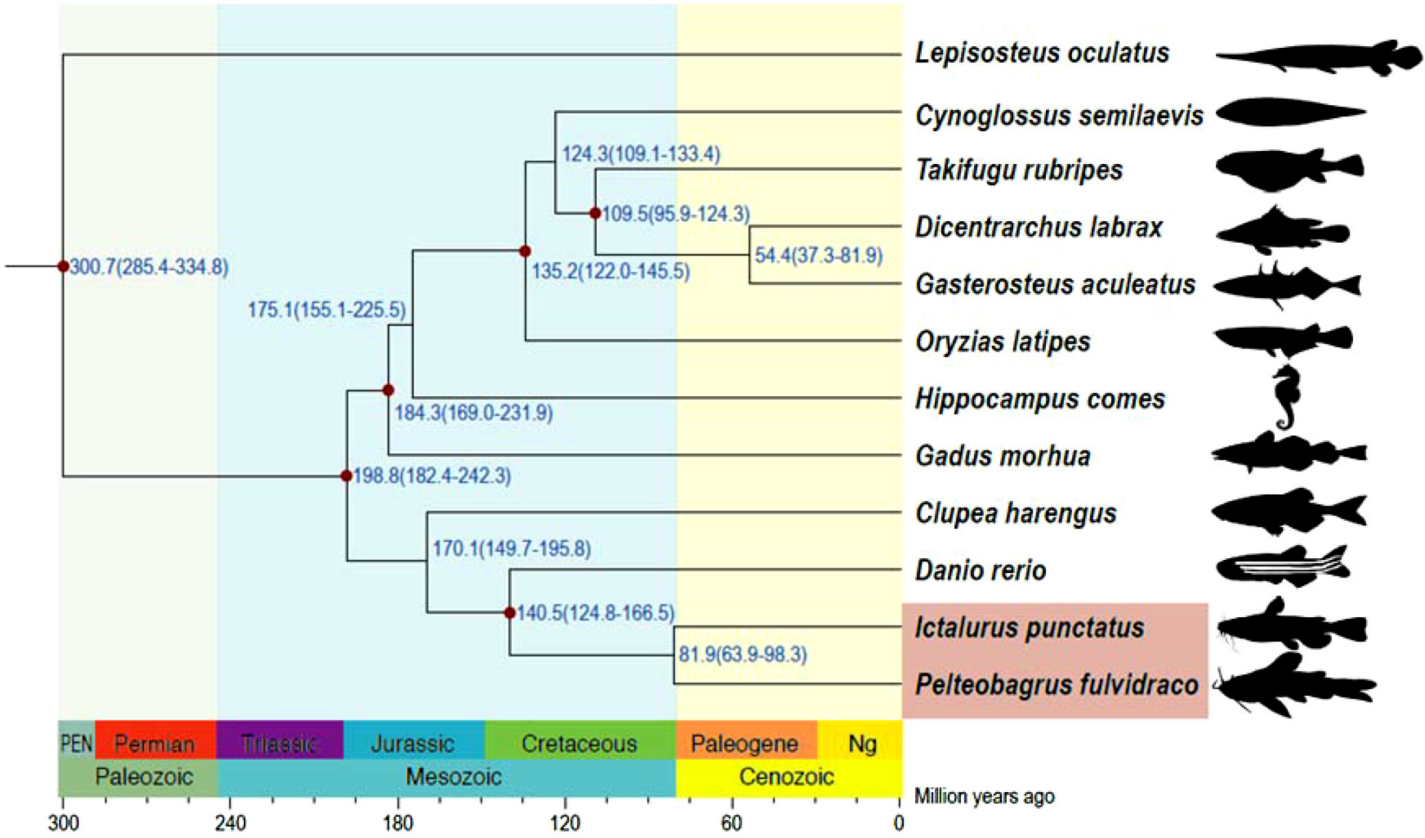
Phylogenetic analysis of the yellow catfish with other teleost species. The estimated species divergence time (million years ago) and the 95% confidential intervals are labeled at each branch site. The divergence used for time recalibration is illuminated as red dots in the tree. The fish (*I. punctatus* and *P. fulvidraco*) from the order Siluriformes are highlighted by pink shading.

To reveal phylogenetic relationships among yellow catfish and other fish species, the protein sequences of single-copy ortholog gene families were aligned with MUSCLE 3.8.31 (RRID:SCR_011812) [44], and the corresponding CDS alignments were generated and concatenated with the guidance of protein alignment. PhyML v3.3 (RRID:SCR_014629) [45] was used to construct the phylogenetic tree for the super-alignment of nucleotide sequences using the JTT+G+F model. Using molecular clock data from the divergence time from the TimeTree database [46], the PAML v4.8 MCMCtree program [47] was employed to determine divergence times with the approximate likelihood calculation method. The phylogenetic relationship of other fish species was consistent with that of previous studies [48]. The phylogenetic analysis based on single-copy orthologs of yellow catfish with other teleosts studied in this work estimated that the yellow catfish speciated around 81.9 million years ago from their common ancestor, the channel catfish (Fig. 5). Given that yellow catfish and channel catfish belong to family Bagridae and Ictaluridae, respectively, the phylogenetic analysis showed that Bagridae and Ictaluridae were separated at a comparable time scale; however, determining the exact time requires more Siluriformes genomes.

### Gene family expansion and contraction analysis

According to divergence times and phylogenetic relationships, CAFE [49] was used to analyze gene family evolution, and 1,717 gene families were significantly expanded in the yellow catfish (*P* < 0.05). The functional enrichment on GO and KEGG of those expanded gene families identified 350 and 42 significantly enriched (q-value < 0.05) GO terms (Supplementary Table S3) and pathways (Supplementary Table S4), respectively. The expanded gene families were mainly found on immune system pathways, especially on hematopoietic cell lineage (q-value = 2.2e-17); the intestinal immune network for immunoglobulin A production (q-value = 2.4e-17); complement and coagulation cascades (q-value = 1.4e-15); antigen processing and presentation (q-value = 2.3e-9) on KEGG pathways; signal transduction pathways, including NF-kappa B signaling pathway (q-value = 5.4e-9), Rap1 signaling pathway (q-value = 1.9e-6), and PI3K-Akt signaling pathway (q-value = 2.3e-4). Meanwhile, 208 GO terms and 44 KEGG pathways, including endocrine system, signal transduction, xenobiotics biodegradation and metabolism, sensory system, were enriched using significantly contracted gene families.

## Conclusion

Combining Illumina and PacBio sequencing platforms with Hi-C technology, we reported the first high-quality chromosome-level genome assembly for the yellow catfish. The contig and scaffold N50 reached 1.1 and 25.8 Mb, respectively. In addition, 24,552 protein-coding genes were identified in the assembled yellow catfish, and 3,088 gene families were clustered for fish species in this work. The phylogenetic analysis of related species showed that yellow catfish diverged ∼81.9 million years ago from the common ancestor of the channel catfish. Expanded gene families were significantly enriched in several important biological pathways, mainly in immune system and signal transduction; important functional genes in those pathways were identified for future studies. Given the economic importance of yellow catfish and the increasing research interests for the species, the genomic data in this work offer a valuable resource for functional gene investigations of yellow catfish. Furthermore, the chromosomal assembly of yellow catfish also provides valuable data for evolutionary studies for the research community, in general.

## Availability of supporting data

The raw sequencing and physical mapping data are available from NCBI via accession numbers SRR7817079, SRR7817060, and SRR7818403 via the project PRJNA489116, as well as the National Omics Data Encyclopedia (http://www.biosino.org/node/index) via project ID OEP000129 (http://www.biosino.org/node/project/detail/OEP000129). The genome, annotation, and intermediate files and results are also available via the *GigaScience* GigaDB repository [50]. All supplementary figures and tables are provided in Supplemental Table S1–S3 and Supplementary Figure S1–S5.

## Software and URLs

**Table:** 

Software	URLs
HTQC	https://sourceforge.net/projects/htqc/
Falcon	https://github.com/PacificBiosciences/FALCON/wiki/Manual
Canu	https://github.com/marbl/canu
GMP	https://github.com/Jianwei-Zhang/LIMS
Pilon	https://github.com/broadinstitute/pilon/
Bowtie	http://bowtie-bio.sourceforge.net/index.shtml
Hiclib	https://bitbucket.org/mirnylab/hiclib/src
Lachesis	https://github.com/shendurelab/LACHESIS
Juicebox	https://www.aidenlab.org/juicebox/
BUSCO	https://busco.ezlab.org/
BWA	http://bio-bwa.sourceforge.net/
GATK	https://software.broadinstitute.org/gatk/
RepeatModeler	http://www.repeatmasker.org/RepeatModeler.html
RepeatMasker	http://repeatmasker.org/
Augustus	https://ngs.csr.uky.edu/Augustus
Balst	https://blast.ncbi.nlm.nih.gov/Blast.cgi
TopHat	https://ccb.jhu.edu/software/tophat/index.shtml
Cufflinks	http://cole-trapnell-lab.github.io/cufflinks/
MAKER	http://www.yandell-lab.org/software/maker.html
Blast2GO	https://www.blast2go.com/
OrthMCL	https://github.com/apetkau/orthomcl-pipeline
MUSCLE	http://www.drive5.com/muscle/
PhyML	https://github.com/stephaneguindon/phyml
TimeTree	http://timetree.org/
PAML	http://abacus.gene.ucl.ac.uk/software/paml.html

## Additional files

Supplementary information_R1.docx.

## Abbreviations

BLAST: Basic Local Alignment Search Tool; BUSCO: Benchmarking Universal Single-Copy Orthologs; CDS: coding DNA sequence; GO: Gene Ontology; GPM: genome puzzle master; KEGG: Kyoto Encyclopedia of Genes and Genomes; NCBI: National Center for Biotechnology Information; NEB: New England Biolabs; PacBio: Pacific Biosciences; RNA-seq: RNA sequencing; SDS: sodium dodecyl sulfate; SNP: single-nucleotide polymorphism; TE: transposable element TE: transposable element.

## Competing interests

The authors declare that they have no competing interests.

## Funding

This work was supported by the China Agriculture Research System (CARS-46) and the Fundamental Research Funds for the Central Universities (2662017PY013).

## Author contributions

J.M., J.-F.G., and N.C. conceived the study; D.C., J.Z., W.G., and P.H. collected the samples and performed sequencing and Hi-C experiments; S.X., G.G., and Y.H. estimated the genome size and assembled the genome; S.X., G.G., and X.L. assessed the assembly quality; G.G., S.X., Y.X., and J.W. carried out the genome annotation and functional genomic analysis; and J.M., N.C., S.X., G.G., and J.-F.G.wrote the manuscript. And all authors read, edited, and approved the final manuscript.

## Supplementary Material

Reviewer_1_Report_(Original_Submission) -- Geoffrey Waldbieser 7/23/2018 Reviewed

Reviewer_2_Report_(Original_Submission) -- Christiaan Henkel 7/29/2018 Reviewed

Reviewer_3_Report_(Original_Submission) -- Paolo Franchini 8/2/2018 Reviewed

Response_To_Reviewer_Comments_(Original_Submission).pdf

GIGA-D-18-00234_Original_Submission.pdf

GIGA-D-18-00234_Revision_1.pdf

Supplemental File
