# Supplementary material for: Chromosomal-level assembly of yellow catfish genome using third-generation DNA sequencing and Hi-C analysis
Source: Gigascience. 2018 Sep 20;7(11):giy120. doi: 10.1093/gigascience/giy120 (PMC6228179; doi:10.1093/gigascience/giy120)
Supplement: GIGA-D-18-00234_Original_Submission.pdf [file giy120_giga-d-18-00234_original_submission.pdf]

GigaScience

Chromosomal-level assembly of yellow catfish genome using third-generation DNA sequencing and Hi-C analysis

--Manuscript Draft--

**Manuscript Number:**	GIGA-D-18-00234
**Full Title:**	Chromosomal-level assembly of yellow catfish genome using third-generation DNA sequencing and Hi-C analysis
**Article Type:**	Data Note
**Funding Information:**	China Agriculture Research System (carS-46) — Dr. Jie Mei<br>the Fundamental Research Funds for the Central Universities (2662017PY013) — Dr. Jie Mei
**Abstract:**	Background<br><br>The yellow catfish, Pelteobagrus fulvidraco, belonging to Siluriformes order, is an important economic freshwater aquaculture fish species in Asia, especially in south China. Recently, the aquaculture industry is facing tremendous challenges in germplasm degeneration and poor diseases resistance. Meanwhile, yellow catfish exhibits notable sex dimorphism on growth rate that adult males are about two to three fold bigger than females. How aquaculture industry takes advantage of such sex dimorphism is another challenge. To address these challenges, a high-quality reference genome of the yellow catfish is needed.<br><br>Finding<br><br>To construct a high-quality reference genome for yellow catfish, we generated 51.2 Gb short reads and 38.9 Gb long reads using Illumina and PacBio platforms, respectively. The sequencing results were assembled into 732.8 Mb genome assembly with contig N50 length of 1.1 Mb. Additionally, we applied Hi-C technology to identify contacts among contigs, which were then used to assemble contigs into scaffolds, resulting in a genome assembly with 26 chromosomes, and a scaffold N50 length of 25.8 Mb. Using 24,552 protein-coding genes annotated in yellow catfish genome, the phylogenetic relationships of yellow catfish with other teleosts showed that yellow catfish separated from the common ancestor of channel catfish ~81.9 million years ago. 1,717 gene families were identified to be expanded in yellow catfish and those gene families are mainly enriched in immune system, signal transduction, glycosphingolipid biosynthesis and fatty acid biosynthesis.<br><br>Conclusion<br><br>Taking advantage of Illumina, PacBio and Hi-C technologies, we constructed the first high-quality chromosomal-level genome assembly for the yellow catfish P. fulvidraco. The genomic resources generated in this work not only offered valuable reference genome for functional genomics studies of yellow catfish to decipher the economic traits and sex determination, but also provided important chromosome information for genome comparisons in broad evolutionary research community.
**Corresponding Author:**	Jie Mei<br>Huazhong Agriculture University<br>Wuhan, Hubei CHINA
**Corresponding Author Secondary Information:**	
**Corresponding Author's Institution:**	Huazhong Agriculture University
**Corresponding Author's Secondary Institution:**	
**First Author:**	Gaorui Gong

Powered by Editorial Manager® and ProduXion Manager® from Aries Systems Corporation

**First Author Secondary Information:**	
**Order of Authors:**	Gaorui Gong
	Cheng Dan
	Shijun Xiao
	Wenjie Guo
	Peipei Huang
	Yang Xiong
	Junjie Wu
	Yan He
	Jicheng Zhang
	Xiaohui Li
	Nansheng Chen
	Jian-Fang Gui
	Jie Mei
**Order of Authors Secondary Information:**	
**Additional Information:**	
**Question**	**Response**
Are you submitting this manuscript to a special series or article collection?	No
**Experimental design and statistics**<br><br>Full details of the experimental design and statistical methods used should be given in the Methods section, as detailed in our Minimum Standards Reporting Checklist. Information essential to interpreting the data presented should be made available in the figure legends.<br><br>Have you included all the information requested in your manuscript?	Yes
**Resources**<br><br>A description of all resources used, including antibodies, cell lines, animals and software tools, with enough information to allow them to be uniquely identified, should be included in the Methods section. Authors are strongly encouraged to cite Research Resource Identifiers (RRIDs) for antibodies, model organisms and tools, where possible.<br><br>Have you included the information requested as detailed in our Minimum Standards Reporting Checklist?	Yes

Powered by Editorial Manager® and ProduXion Manager® from Aries Systems Corporation

**Availability of data and materials**<br><br>All datasets and code on which the conclusions of the paper rely must be either included in your submission or deposited in publicly available repositories (where available and ethically appropriate), referencing such data using a unique identifier in the references and in the "Availability of Data and Materials" section of your manuscript.<br><br>Have you have met the above requirement as detailed in our Minimum Standards Reporting Checklist?	Yes

Powered by Editorial Manager® and ProduXion Manager® from Aries Systems Corporation

Click here to download Manuscript submit_giga_20160621.docx

Chromosomal-level assembly of yellow catfish genome using third-generation DNA sequencing and Hi-C analysis

Gaorui Gong[1,#], Cheng Dan[1,#], Shijun Xiao[2,#], Wenjie Guo[1,#], Peipei Huang[3], Yang Xiong[1], Junjie Wu[1], Yan He[1], Jicheng Zhang[2], Xiaohui Li[1], Nansheng Chen[4,5,*], Jian-Fang Gui[1,3,*], Jie Mei[1,*]

[1] College of Fisheries, Key Laboratory of Freshwater Animal Breeding, Ministry of Agriculture, Huazhong Agricultural University, Wuhan, China.

[2] Wuhan Frasergen Bioinformatics, East Lake High-Tech Zone, Wuhan, China.

[3] State Key Laboratory of Freshwater Ecology and Biotechnology, Institute of Hydrobiology, Chinese Academy of Sciences, University of the Chinese Academy of Sciences, Wuhan, China.

[4] Institute of Oceanology, Chinese Academy of Sciences, Qingdao, Shandong, China

[5] Department of Molecular Biology and Biochemistry, Simon Fraser University, Burnaby, Canada

[#] These authors contributed equally to this work.

***** Corresponding author. Tel: +86-27-87282113; Fax: +86-27-87282114.

Email address: jmei@mail.hzau.edu.cn (Dr. Jie Mei)

jfgui@ihb.ac.cn (Dr. Jian-Fang Gui)

chenn@sfu.ca (Dr. Nansheng Chen)

Abstract

Background: The yellow catfish, *Pelteobagrus fulvidraco*, belonging to Siluriformes order, is an important economic freshwater aquaculture fish species in Asia, especially in south China. Recently, the aquaculture industry is facing tremendous challenges in germplasm degeneration and poor diseases resistance. Meanwhile, yellow catfish exhibits notable sex dimorphism on growth rate that adult males are about two to three fold bigger than females. How aquaculture industry takes advantage of such sex dimorphism is another challenge. To address these challenges, a high-quality reference genome of the yellow catfish is needed.

Finding: To construct a high-quality reference genome for yellow catfish, we generated 51.2 Gb short reads and 38.9 Gb long reads using Illumina and PacBio platforms, respectively. The sequencing results were assembled into 732.8 Mb genome assembly with contig N50 length of 1.1 Mb. Additionally, we applied Hi-C technology to identify contacts among contigs, which were then used to assemble contigs into scaffolds, resulting in a genome assembly with 26 chromosomes, and a scaffold N50 length of 25.8 Mb. Using 24,552 protein-coding genes annotated in yellow catfish genome, the phylogenetic relationships of yellow catfish with other teleosts showed that yellow catfish separated from the common ancestor of channel catfish ~81.9 million years ago. 1,717 gene families were identified to be expanded in yellow catfish and those gene families are mainly enriched in immune system, signal transduction, glycosphingolipid biosynthesis and fatty acid biosynthesis.

Conclusion: Taking advantage of Illumina, PacBio and Hi-C technologies, we constructed the first high-quality chromosomal-level genome assembly for the yellow catfish *P. fulvidraco*. The genomic resources generated in this work not only offered valuable reference genome for functional genomics studies of yellow catfish to decipher the economic traits and sex determination, but also provided important chromosome information for genome comparisons in broad evolutionary research community.

Key Words: yellow catfish, PacBio, Hi-C, chromosomal assembly

Data description

Introduction

The yellow catfish, *Pelteobagrus fulvidraco*, (Richardson, 1846; NCBI Taxonomy ID: 1234273) is a teleost fish belonging to the order Siluriformes, and is an economically important freshwater fish species in Asia.[1] In recent years, yellow catfish has become one of the most important aquaculture species in China with an increasing market value in aquaculture industry because of its high meat quality. However, since the ultra-dense aquaculture and the loss of genetic diversity, artificial breeding of yellow catfish is facing tremendous challenges such as germplasm degeneration and poor diseases resistance.[2] Meanwhile, yellow catfish is also an excellent model for studying sex differentiation and sexual evolution in fish species[3,4], since female and male yellow catfish exhibited remarkable sex dimorphism on growth rate that adult yellow catfish males are about two to three fold bigger than the females. In the last decade, sex-specific allele marker were developed and YY super-male fish were generated from gynogenesis of XY physiological female fish.[1,5]

In spite of the importance of yellow catfish both in sex-determination research and in aquaculture, the genomic resources for the species is still limited. So far, only transcriptome, SSR and SNP data were reported for yellow catfish in previous studies[4], the genome sequence for this important species is still missing, hindering the genome-based functional gene identification controlling important economic traits and the application of genome-assisted breeding in yellow catfish. In this work, we combined genomic sequencing data from Illumina short reads and PacBio long reads to generate the first reference genome for yellow catfish, and applied Hi-C data to scaffold the genome sequences into the chromosomal level. The completeness and continuity of the genome were comparable with other model teleost species. We believe that the high-quality reference genome generated in this work will definitely facilitate research on population genetics and functional genes identification related to important economic traits and the sex determinant for yellow catfish, which will in turn accelerate the development of more efficient sex control techniques and improve the artificial breeding industry for this important economical fish species.

Sample and sequencing

A female yellow catfish, reared in the breeding center of Huazhong Agricultural University in Wuhan City, Hubei Province, was used for preparing DNA for sequencing. To obtained sufficient high-quality DNA molecules for PacBio Sequel platform (Pacific Biosciences of California, Menlo Park, CA, USA), one yellow catfish was dissected and fresh muscle

tissues were used for DNA extraction using phenol/chloroform extraction method. The quality of the DNA was checked by agarose gel electrophoresis, and an excellent integrity of DNA molecules were observed. Other tissues, including ocular, skin, muscle, gonadal, intestinal, liver, kidney, blood, gall and air bladder tissues were quickly frozen in liquid nitrogen for at one hour and then stored at −80 °C.

The extracted DNA molecules were sequenced with both Illumina HiSeq X Ten platform (Illumina Inc., San Diego, CA, USA) and PacBio Sequel platform. Short reads generated from Illumina platform were used for genome characters evaluation, and long reads from PacBio platform were used for genome assembly. To this end, one library with an insertion length of 250 bp was generated for HiSeq X Ten platform and three 20 kb libraries were constructed for PacBio platform according to the according to manufacturer's protocol, resulting the generation of ~51.2 Gb short reads and ~38.9 Gb long reads, respectively. The polymerase and subreads N50 length reached21.3 kb and 16.2 kb, providing ultra-long genomic sequences for the following assembly.

Genome features estimation from *K*mer method

The short-reads from Illumina platform were quality filtered by HTQC[6] with the method as follows. First, the adaptors were removed from the sequencing reads. Second, read pairs were excluded if any one end has an average quality lower than 20.Third, ends of reads were trimmed if the average quality lower than 20 in the sliding window size of 5 bp. Finally, read pairs with any end was shorter than 75 bp were removed.

The quality filtered reads were used for genome size estimation. Using the *K*mer method described in previous method[7], we calculated and plot the 17-mer depth distribution in SI Figure 1. The formula $G = N_{17\text{-mer}} / D_{17\text{-mer}}$,where the $N_{17\text{-mer}}$ is the total number of 17-mers,and $D_{17\text{-mer}}$ denotes the peak frequency of 17-mers, were used to estimate the genome size of yellow catfish. As a result, we estimated genome size of 712 Mb. Meanwhile, a heterozygosity of 0.45% and repeat ratio of 43.31%.

Genome assembly by third-generation long reads

With 6 flow cells in PacBio Sequel platform, we generated 38.9 Gb subreads by removing adaptor sequences within sequences. The mean and N50 length were 9.8 and 16.2 kb, respectively. The long subreads were used for genomic assembly of yellow catfish. Firstly, Falcon package[8] with a parameter of length_cutoff as 10 kb and pr_length_cutoff as 8 kb. As a result, we obtained a 690 Mb genome with a contig N50 length of 193.1 kb. Secondly, canu v1.5[9] was employed separately for genome assembly with default parameters, leading to 688.6 Mb yellow catfish genome with contig N50 of 427.3kb. Although the size of genome assembly from both Falcon and canu was comparable with the estimation based on *K*mer method, the continuity of the genome need further improvement. Taking

advantage of the sequence complementation of the two assemblies, we therefore applied Genome Puzzle Master (GPM)[10] to merge long contigs using reliable overlaps between sequences. Finally, a ~730 Mb genome assembly of yellow catfish with 3,564 contigs and contig N50/L50 of 1.1 Mb/126 was constructed. The final genome sequences were then polished by arrow[11] using PacBio long reads and by plion[12] using Illumina short reads to correct errors in base level.

In situ Hi-C library construction and chromosome assembly using Hi-C data

Blood sample of yellow catfish was used for library construction for Hi-C analysis. 0.1ml blood were used for Hi-C library construction. The extracted nuclei were re-suspended with 150 μl 0.1% SDS and split equally into three tubes. The nuclei were incubated at 65°C for 10 min, after the SDS molecules were quenched by adding 120 μl water and 30 μl 10% Triton X-100, and incubated at 37 °C for 15 min. The DNA in the nuclei in each tube was digested by adding 30 μl 10x NEB buffer 2.1(50 mM NaCl, 10 mM Tris-HCl, 10 mM MgCl2, 100 μg/ml BSA, pH 7.9) and 150U of MboI, and incubated at 37 °C overnight. On the next day, the MboI enzyme was inactivated at 65 °C for 20 min. Next, the cohesive ends were filled in by adding 1 μl of 10 mM dTTP, 1μl of 10 mM dATP, 1 μl of 10 mM dGTP, 2 μl of 5mM biotin-14-dCTP, 14 μl water and 4 μl (40 U) Klenow, and incubated at 37 °C for 2 h. Subsequently, 663 μl water,120 μl 10x blunt-end ligation buffer (300 mM Tris-HCl, 100 mM $MgCl_2$, 100 mM DTT, 1 mM ATP, pH 7.8), 100μl 10% Triton X-100 and 20 U T4 DNA ligase were added to start proximity ligation. The ligation reaction was placed at 16 °C for 4 h. Next, the reaction mixture was centrifuged at 1000 g for 3 min, and the nuclei pellet was re-suspended with 750 μl SDS buffer (50 mM Tris-HCl, 1% SDS, 10 mM EDTA, pH 8.0) and incubated with 200 μg proteinase K at 65 °C for 4 h. The formaldehyde cross-link was reversed by adding 30 μl 5 M NaCl to the solution followed by incubation at 65 °C overnight. Subsequent chromatin DNA manipulations were performed as described in previous study. The final library was sequenced on the Illumina HiSeq X Ten platform (San Diego, CA, United States) with 150PEmode.

487 million raw reads were generated from the Hi-C library and were mapped to the polished yellow catfish genome using Bowtie (RRID:SCR_005476)[13] with the default parameters. The iterative method was used to increase the interactive Hi-C reads ratio[14]. Two ends of paired reads were mapped to the genome independently, but only the reads that two pairs were uniquely mapped to genome were used. Self-ligation, non-ligation and other invalid reads, such as StartNearRsite, PCR amplification, random break, LargeSmallFragments and ExtremeFragments, were filtered using the method and Hi-Clib as described in previous reports. The contact count among each contig were calculated and normalized by the restriction sites in sequences (Figure 2). We then successfully clustered 2,965 contigs into 26 groups with the agglomerative hierarchical clustering method in Lachesis[15], which was consistent with the previous karyotype analyses of

Pseudobagrus fulvidraco[16]. Lachesis was further applied to order and orient the clustered contigs, and 2,440 contigs were reliably anchored on chromosomes, presenting 66.8% and 94.2% of the total genome by sequence number and base count, respectively. Then, we applied juicebox[17] to correct the contig orientation and to remove suspicious fragments in contig to unanchored groups by visual inspection. Finally, we obtained the first chromosomal-level high-quality yellow catfish assembly with a contig N50 of 1.1 Mb and scaffold N50 of 25.8 Mb, providing solid genomic resource for the following population and functional analysis.

Genome quality evaluation

First of all, we compared the genome assembly continuity of the yellow catfish genome to those of other teleost species. We found that both contig and scaffold N50 length of yellow catfish reached considerable continuity (Figure 3), providing us a high-quality genome sequences for the following functional investigations. The assembled genome were also subjected the BUSCO[18] (RRID:SCR_015008, version 3.0) to evaluate the completeness of the genome. We identified 91.2% BUSCO genes in yellow catfish genome. After aligning short reads from Illumina platform to the genome, the insertion length distribution for sequencing library of 250 bp exhibited a single peak around the sequencing library length design (SI Figure 2). Paired-end reads data were not used during the contig assembly, thus the high alignment ratio and single peak insertion length distribution demonstrated the high-quality of contig assembly for yellow catfish. Using the Illumina short read alignment, we have identified 21,143 homologous SNP loci by GATK (RRID:SCR_001876) package[19], suggesting that the accuracy of our genome reached upto 99.997% on base level.

Repeat and gene annotation

We first used Tandem Repeat Finder[20] to identify repetitive elements in yellow catfish genome. RepeatModeler (http://www.repeatmasker.org/RepeatModeler.html, RRID:SCR_015027) were used to detect transposon elements (TE) in the genome by a *de novo* manner. The *de novo* and known repeats library from Repbase[21] were then combined, and the TEs were detected by mapping sequences to the combined library in yellow catfish genome using the software RepeatMasker (RRID:SCR_012954)[22].

For protein-coding gene annotation, *de novo*-, homology- and RNA-seq-based methods were used. Augustus (RRID:SCR_008417)[23] was used to predict-coding genes in *de novo* prediction. For homology-based method, protein sequences of closely related fish species, including *Astyanax mexicanus*, *Danio rerio*, *Gadus morhua*, *Ictalurus punctatus, Oryzias latipes*，*Takifugu rubripes*，*Tetraodon nigroviridis* and *Oreochromis niloticus* were downloaded from Ensembl[24] and were aligned against to the yellow catfish genome using TBLASTN (RRID:SCR_011822) software[25]. Short reads from RNA-Seq

(SRR1845493) were also mapped upon the genome using TopHat (RRID:SCR_013035) package[26], and the gene structure were formed using Cufflinks (RRID:SCR_014597)[27]. Finally, 24,552 consensus protein-coding genes were predicted in the yellow catfish genome by integrating all gene models by MAKER[28]. The gene number, gene length distribution, CDS length distribution, exon length distribution and intron length distribution were comparable with those in other teleost fish species (SI Figure 3).

Local BLASTX (RRID:SCR_001653) and BLASTN (RRID:SCR_001598) programs were used to search all predicted gene sequences to NCBI non-redundant protein (nr), non-redundant nucleotide (nt), Swissprot database with an e-value of 1e-5[29]. Gene ontology (GO)[30] and Kyoto Encyclopedia of Genes and Genomes (KEGG)[31] pathway annotation were also assigned to genes using the software Blast2GO[32].As a result, 24,552 genes were annotated to at least one database. (Table 2)

Gene family identification and Phylogenetic analysis of yellow catfish

To cluster families from protein-coding gene, proteins from the longest transcripts of each genes from yellow catfish and other fish species, including *Ictalurus punctatus*, *Clupeaharengus*, *Danio rerio*, *Takifugu rubripes*, *Hippocampus comes*, *Cynoglossus semilaevis*, *Oryzias latipes*, *Gadus morhua*, *Lepisosteus oculatus*, *Dicentrarchus labrax*, and *Gasterosteus aculeatus,* were extracted andaligned to each other with BLASTP (RRID:SCR_001010) programs[29] with an evalue of 1e-5. OrthMCL[33] was used to cluster gene family using protein BLAST result. As a result, 19,846 gene families were constructed for fish species in this work and 3,088 families were identified as single-copy ortholog gene families.

To reveal phylogenetic relationships among yellow catfish and other fish species, the protein sequences of single-copy ortholog gene family were aligned with MUSCLE (RRID:SCR_011812) program[34], and the corresponding Coding DNA Sequences (CDS) alignments were generated and concatenated with the guidance of protein alignment. PhyML (RRID:SCR_014629)[35] were used to construct the phylogenetic tree for the super-alignment of nucleotide sequences with JTT+G+F model. Using molecular clock data from the divergence time from the TimeTree database[36], the PAML MCMCtree program[37] was employed to determine divergence times with the approximate likelihood calculation method. The phylogenetic analysis based on single-copy orthologs of yellow catfish with other teleosts studied in this work estimated that the yellow catfish speciated around 81.9 million years ago from their common ancestor of the channel catfish (Figure 4). Given yellow catfish and channel catfish belong to family Bagridae and Ictaluridae respectively[38], the phylogenetic analysis showed that Bagridae and Ictaluridae were separated at the comparable time scale, however, the exact time estimation need more genomes in Siluriformes.

Gene family expansion and contraction analysis

According to divergence times and phylogenetic relationships, CAFE[39] was used to analyze gene family evolution and 1,717 gene families were significantly expanded in the yellow catfish (P < 0.05). The functional enrichment on GO and KEGG of those expanded gene families identified 350 and 42 significantly enriched (q-value < 0.05) GO terms (**SI Table 1**) and pathways (**SI Table 2**), respectively. The expanded gene families were mainly found on immune system pathways, especially on Hematopoietic cell lineage (q-value = 2.2e-17), Intestinal immune network for IgA production (q-value = 2.4e-17), Complement and coagulation cascades (q-value = 1.4e-15) and Antigen processing and presentation (q-value = 2.3e-9) on KEGG pathways, and Signal transduction pathways, including NF-kappa B signaling pathway (q-value = 5.4e-9), Rap1 signaling pathway (q-value = 1.9e-6) and PI3K-Akt signaling pathway (q-value = 2.3e-4). Meanwhile, 208 GO terms and 44 KEGG pathways, including endocrine system, signal transduction, xenobiotics biodegradation and metabolism, sensory system were enriched using significantly contracted gene families.

Conclusion

Using third-generation PacBio Sequel sequencing platform and Hi-C technology, we reported the first high-quality chromosomal level genome assembly for yellow catfish. The contig and scaffold N50 reached 1.1 and 25.8 Mb, respectively. 24,552 protein-coding were identified in the assembled yellow catfish, and 3,088 gene families were clustered for fish species in this work. The phylogenetic analysis of related species showed that yellow catfish were diverged ~81.9 MYA from the common ancestor of the channel catfish. Expanded gene families were significantly enriched in several important biological pathways, mainly in immune system and signal transduction, and important functional gene in those pathways were identified for following studies. Given the economic importance of yellow catfish and the increasing research interests for the species, the genomic data in this work offered valuable resource for functional gene investigations of yellow catfish. Meanwhile, the chromosomal assembly of yellow catfish also provided valuable data for evolutionary studies for the research community in general.

Availability of supporting data

The raw sequencing and physical mapping data were deposited into The National Omics Data Encyclopedia (NODE) (http://www.biosino.org/node/index) with the project ID of OEP000129 (http://www.biosino.org/node/project/detail/OEP000129). The genome, annotation and intermediate files were uploaded to GigaScience FTP server. All

supplementary figures and tables are provided in Supplemental File.

Competing interests

The authors declare that they have no competing interests.

Funding

This work was supported by China Agriculture Research System (CARS-46) and the Fundamental Research Funds for the Central Universities (2662017PY013).

Author Contributions

Jie Mei, Jian-Fang Gui and Nansheng Chen conceived the study; Dan Chen, Jicheng Zhang, Wenjie Guo and Peipei Huang collected the samples and performed sequencing and Hi-C experiments; Shijun Xiao, Gaorui Gong and Yan He estimated the genome size and assembled the genome; Shijun Xiao, Gaorui Gong and Xiaohui Li assessed the assembly quality; Gaorui Gong, Shijun Xiao, Yang Xiong and Junjie Wu carried out the genome annotation and functional genomic analysis, Jie Mei, Nansheng Chen, Shijun Xiao, Gaorui Gong and Jian-Fang Gui wrote the manuscript. And all authors read, edited, and approved the final manuscript.

References

1 Liu, H. *et al.* Genetic manipulation of sex ratio for the large-scale breeding of YY super-male and XY all-male yellow catfish (Pelteobagrus fulvidraco (Richardson)). *Marine Biotechnology* **15**, 321-328 (2013).

2 Liu, F. *et al.* Effects of astaxanthin and emodin on the growth, stress resistance and disease resistance of yellow catfish (Pelteobagrus fulvidraco). *Fish & Shellfish Immunology* **51**, 125 (2016).

3 Jie, M. & Gui, J. F. Genetic basis and biotechnological manipulation of sexual dimorphism and sex determination in fish. *Science China Life Sciences* **58**, 124 (2015).

4 Chen, X. *et al.* A comprehensive transcriptome provides candidate genes for sex determination/differentiation and SSR/SNP markers in yellow catfish. *Marine Biotechnology* **17**, 190-198 (2015).

5 Dan, C., Mei, J., Wang, D. & Gui, J. F. Genetic Differentiation and Efficient Sex-specific Marker Development of a Pair of Y- and X-linked Markers in Yellow Catfish. *International Journal of Biological Sciences* **9**, 1043-1049 (2013).

6 Yang, X. *et al.* HTQC: a fast quality control toolkit for Illumina sequencing data. *Bmc Bioinformatics* **14**, 1-4 (2013).

7 Xu, P. *et al.* Genome sequence and genetic diversity of the common carp, Cyprinus carpio. *Nature Genetics* **46**, 1212-1219 (2014).

8 Chin, C. S. *et al.* Phased Diploid Genome Assembly with Single Molecule Real-Time Sequencing. *Nature Methods* **13**, 1050 (2016).

9 Koren, S. *et al.* Canu: scalable and accurate long-read assembly via adaptive k-mer weighting and repeat separation. *Genome Research* **27**, 722 (2017).

10 Zhang, J. *et al.* in *International Plant and Animal Genome Conference Xx.*

11 Chin, C. S. *et al.* Nonhybrid, finished microbial genome assemblies from long-read SMRT sequencing data. *Nature Methods* **10**, 563 (2013).

12 Walker, B. J. *et al.* Pilon: An Integrated Tool for Comprehensive Microbial Variant Detection and Genome Assembly Improvement. *Plos One* **9**, e112963 (2014).

13 Langmead, B., Trapnell, C., Pop, M. & Salzberg, S. L. Ultrafast and memory-efficient alignment of short DNA sequences to the human genome. *Genome Biology* **10**, R25 (2009).

14 Nicolas, S. *et al.* HiC-Pro: an optimized and flexible pipeline for Hi-C data processing. *Genome Biology* **16**, 259 (2015).

15 Burton, J. N. *et al.* Chromosome-scale scaffolding of de novo genome assemblies based on chromatin interactions. *Nature Biotechnology* **31**, 1119-1125 (2013).

16 Shu-qun, X. Karyotype analyses of Pseudobagrus fulvidraco. *Chinese Journal of Fisheries* (2006).

17 Dudchenko, O. *et al.* The Juicebox Assembly Tools module facilitates de novo assembly of mammalian genomes with chromosome-length scaffolds for under $1000. (2018).

18 Simão, F. A., Waterhouse, R. M., Ioannidis, P., Kriventseva, E. V. & Zdobnov, E. M. BUSCO: assessing genome assembly and annotation completeness with single-copy orthologs. *Bioinformatics* **31**, 3210 (2015).

19 Mckenna, A. *et al.* The Genome Analysis Toolkit: A MapReduce framework for analyzing next-generation DNA sequencing data. *Genome Research* **20**, 1297-1303 (2010).

20 Benson, G. Tandem repeats finder: a program to analyze DNA sequences. *Nucleic Acids Research* **27**, 573 (1999).

21 Bao, W., Kojima, K. K. & Kohany, O. Repbase Update, a database of repetitive elements in eukaryotic genomes. *Mobile Dna* **6**, 11 (2015).

22 Chen, N. Using RepeatMasker to identify repetitive elements in genomic sequences. *Current Protocols in Bioinformatics* **Chapter 4**, Unit 4.10 (2004).

23 Stanke, M. *et al.* AUGUSTUS: ab initio prediction of alternative transcripts. *Nucleic Acids Research* **34**, 435-439 (2006).

24 Flicek, P. *et al.* Ensembl 2014. *Nucleic Acids Research* **42**, D749-D755 (2014).

25 Gertz, E. M. *et al.* Composition-based statistics and translated nucleotide searches: Improving the TBLASTN module of BLAST. *Bmc Biology* **4**, 41 (2006).

26 Trapnell, C., Pachter, L. & Salzberg, S. L. TopHat: discovering splice junctions with RNA-Seq. *Bioinformatics* **25**, 1105-1111 (2009).

27 Ghosh, S. & Chan, C. K. K. Analysis of RNA-Seq Data Using TopHat and Cufflinks. *Methods in Molecular Biology* **1374**, 339 (2016).

28 Campbell, M. S., Holt, C., Moore, B. & Yandell, M. Genome Annotation and Curation Using

MAKER and MAKER-P. *Current Protocols in Bioinformatics* **48**, 4.11.11 (2014).

29 Altschul, S. F., Gish, W., Miller, W., Myers, E. W. & Lipman, D. J. Basic local alignment search tool. *Journal of Molecular Biology* **215**, 403-410 (1990).

30 Harris, M. A. *et al.* The Gene Ontology (GO) database and informatics resource. *Nucleic Acids Research* **32**, D258-261, doi:10.1093/nar/gkh036 (2004).

31 Ogata, H. *et al.* KEGG: Kyoto Encyclopedia of Genes and Genomes. *Nucleic Acids Research* **27**, 29-34 (2000).

32 Conesa, A. *et al.* Blast2GO: a universal tool for annotation, visualization and analysis in functional genomics research. *Bioinformatics* **21**, 3674 (2005).

33 Li, L., Stoeckert, C. J. & Roos, D. S. OrthoMCL: Identification of Ortholog Groups for Eukaryotic Genomes. *Genome Research* **13**, 2178-2189 (2003).

34 Thompson, J. D., Gibson, T. J. & Higgins, D. G. *Multiple Sequence Alignment Using ClustalW and ClustalX*. (John Wiley & Sons, Inc., 2002).

35 Guindon, S., Dufayard, J. F., Hordijk, W., Lefort, V. & Gascuel, O. PhyML: Fast and Accurate Phylogeny Reconstruction by Maximum Likelihood. **9**, 384-385 (2009).

36 Hedges, S. B., Dudley, J. & Kumar, S. TimeTree: a public knowledge-base of divergence times among organisms. *Bioinformatics* **22**, 2971-2972 (2006).

37 Yang, Z. PAML: a program package for phylogenetic analysis by maximum likelihood. *Computer Applications in Bioscience* **13**, 555-556 (1997).

38 Liu, Z. *et al.* The channel catfish genome sequence provides insights into the evolution of scale formation in teleosts. *Nature Communications* **7**, 11757 (2016).

39 De Bie, T., Cristianini, N., Demuth, J. P. & Hahn, M. W. CAFE: a computational tool for the study of gene family evolution. *Bioinformatics* **22**, 1269-1271 (2006).

Tables and Figures

Tables

Table 1 Sequencing data generated for yellow catfish genome assembly and annotation

Library type	method	Library size (bp)	Data size (Gb)	Application
**DNA**	HiSeq XTen	250	51.2	genome survey and genomic base correction
**DNA**	PacBio SEQUEL	20,000	38.9	genome assembly
**Hi-C**	HiSeq XTen	250	146.1	chromosome construction

Table 2. Statistics for genome annotation of yellow catfish

Database	Number	Percent
InterPro	20,178	82.18
GO	14,936	60.83
KEGG ALL	24,025	97.85
KEGG KO	13,951	56.82
Swissprot	20,875	85.02
TrEMBL	24,093	98.13
NR	24,308	99.01
Total	24,552	

Figures

Figure 1. A picture of yellow catfish.

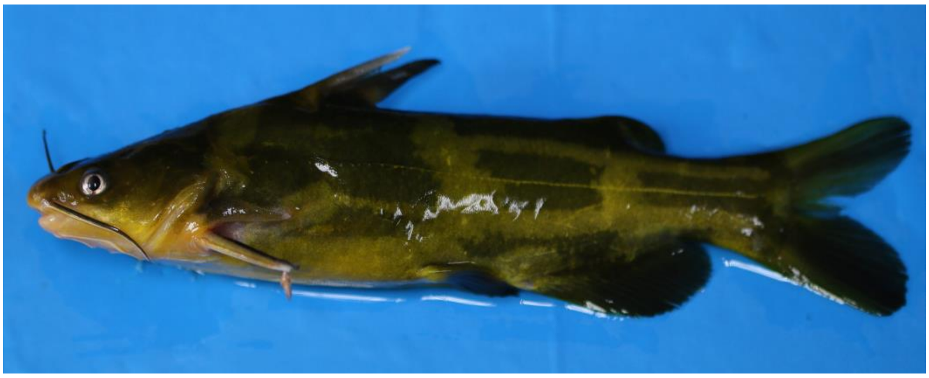

Figure 2. Yellow catfish genome contig contact matrix using Hi-C data.

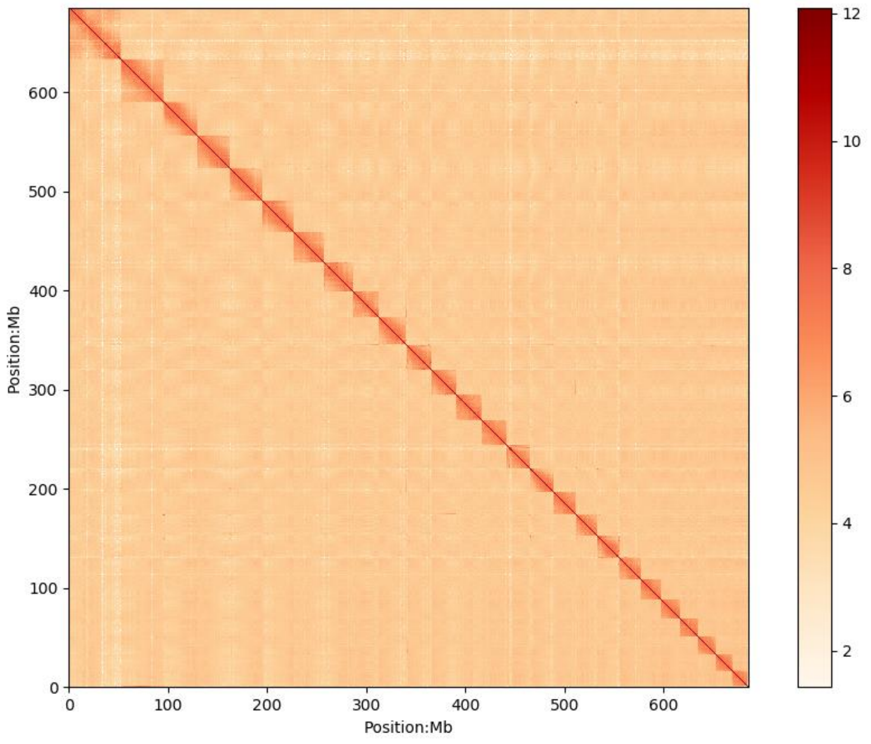

Figure 3. Genome assembly comparison of yellow catfish with other public teleost genomes.

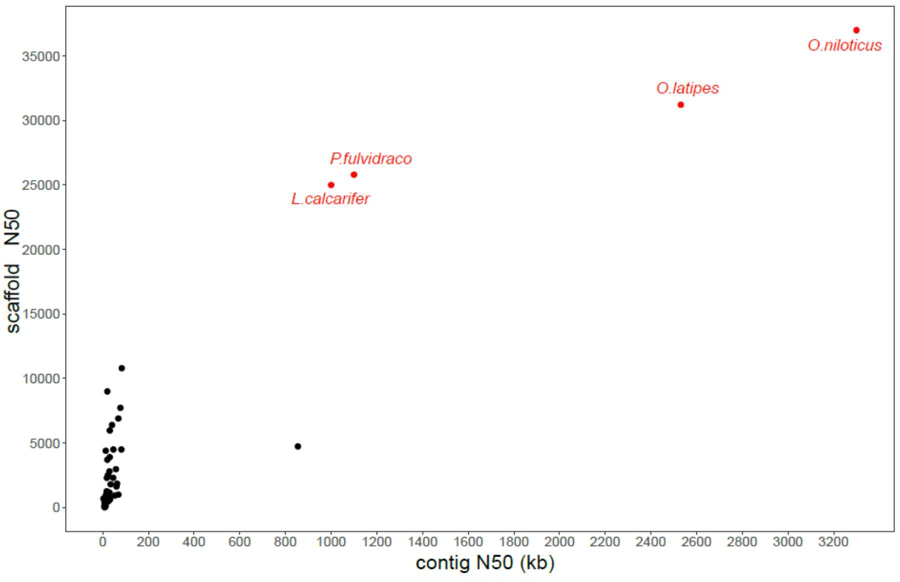

Figure 4. Phylogenetic analysis of yellow catfish with other teleost species.

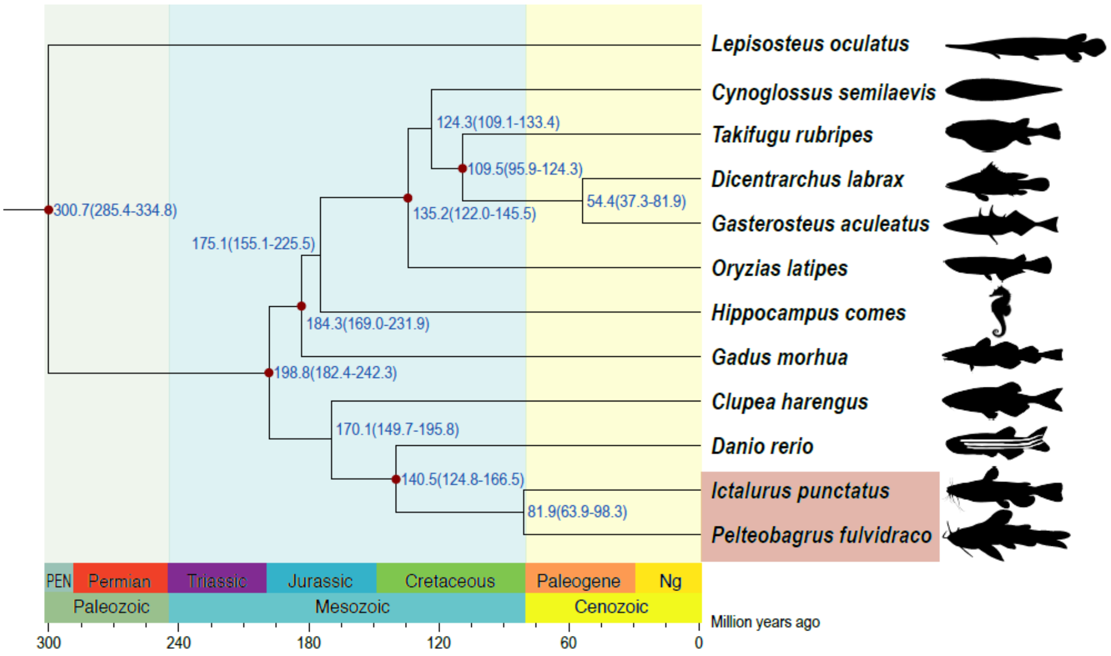

Supplementary Figure 1

Click here to access/download
Supplementary Material
Supplyementary Figure1.pdf

Supplementary Figure 2

Click here to access/download
Supplementary Material
Supplyementary Figure2.pdf

Supplementary Figure 3

Click here to access/download
Supplementary Material
Supplyementary Figure3.pdf

Supplementary Table 1

Click here to access/download

Supplementary Material

Supplemenatary Table1_GO_enerichment.xlsx

Supplementary Table 2

Click here to access/download
Supplementary Material
Supplemenatary Table2_KEGG_enerichment.xlsx
