# Supplementary material for: Chromosomal-level assembly of yellow catfish genome using third-generation DNA sequencing and Hi-C analysis
Source: Gigascience. 2018 Sep 20;7(11):giy120. doi: 10.1093/gigascience/giy120 (PMC6228179; doi:10.1093/gigascience/giy120)
Supplement: GIGA-D-18-00234_Revision_1.pdf [file giy120_giga-d-18-00234_revision_1.pdf]

GigaScience

Chromosomal-level assembly of yellow catfish genome using third-generation DNA sequencing and Hi-C analysis

--Manuscript Draft--

**Manuscript Number:**	GIGA-D-18-00234R1
**Full Title:**	Chromosomal-level assembly of yellow catfish genome using third-generation DNA sequencing and Hi-C analysis
**Article Type:**	Data Note
**Funding Information:**	China Agriculture Research System (carS-46) — Dr. Jie Mei<br>the Fundamental Research Funds for the Central Universities (2662017PY013) — Dr. Jie Mei
**Abstract:**	Background: The yellow catfish, Pelteobagrus fulvidraco, belonging to Siluriformes order, is an economically important freshwater aquaculture fish species in Asia, especially in Southern China. The aquaculture industry has recently been facing tremendous challenges in germplasm degeneration and poor diseases resistance. As the yellow catfish exhibits notable sex dimorphism in growth, with adult males about two to three fold bigger than females, how aquaculture industry takes advantage of such sex dimorphism is another challenge. To address these issues, a high-quality reference genome of the yellow catfish would be a very useful resource.<br>Finding: To construct a high-quality reference genome for the yellow catfish, we generated 51.2 Gb short reads and 38.9 Gb long reads using Illumina and PacBio sequencing platforms, respectively. The sequencing data were assembled into a 732.8 Mb genome assembly with a contig N50 length of 1.1 Mb. Additionally, we applied Hi-C technology to identify contacts among contigs, which were then used to assemble contigs into scaffolds, resulting in a genome assembly with 26 chromosomes, and a scaffold N50 length of 25.8 Mb. Using 24,552 protein-coding genes annotated in the yellow catfish genome, the phylogenetic relationships of the yellow catfish with other teleosts showed that yellow catfish separated from the common ancestor of channel catfish ~81.9 million years ago. 1,717 gene families were identified to be expanded in the yellow catfish and those gene families are mainly enriched in immune system, signal transduction, glycosphingolipid biosynthesis and fatty acid biosynthesis.<br>Conclusion: Taking advantage of Illumina, PacBio and Hi-C technologies, we constructed the first high-quality chromosomal-level genome assembly for the yellow catfish P. fulvidraco. The genomic resources generated in this work not only offer a valuable reference genome for functional genomics studies of yellow catfish to decipher the economic traits and sex determination, but also provide important chromosome information for genome comparisons in the wider evolutionary research community.
**Corresponding Author:**	Jie Mei<br>Huazhong Agriculture University<br>Wuhan, Hubei CHINA
**Corresponding Author Secondary Information:**	
**Corresponding Author's Institution:**	Huazhong Agriculture University
**Corresponding Author's Secondary Institution:**	
**First Author:**	Gaorui Gong
**First Author Secondary Information:**	
**Order of Authors:**	Gaorui Gong
	Cheng Dan
	Shijun Xiao

Powered by Editorial Manager® and ProduXion Manager® from Aries Systems Corporation

	Wenjie Guo
	Peipei Huang
	Yang Xiong
	Junjie Wu
	Yan He
	Jicheng Zhang
	Xiaohui Li
	Nansheng Chen
	Jian-Fang Gui
	Jie Mei
**Order of Authors Secondary Information:**	
**Response to Reviewers:**	Dear Editors:<br><br>We are submitting the revised manuscript entitled "Chromosomal-level assembly of yellow catfish genome using third-generation DNA sequencing and Hi-C analysis". You can find our detailed answers to points raised by reviewers in our response letter. We have revised our manuscript according to the comments from the reviewers.<br><br>You and other reviewers are high appreciated for your constructive suggestions. We believe the quality and scientific significance of the manuscript has improved greatly due to the feedback of the reviewers. We hope that the paper is now in a form suitable for publication in Giga Science as a Data Note.<br><br>Yours sincerely,<br><br>Prof. Dr. Jie Mei<br>College of Fisheries<br>Huazhong Agricultural University, Wuhan, China<br>Email: jmei@mail.hzau.edu.cn<br>Aug 22, 2018<br><br>Reviewer reports:<br>Reviewer #1: This manuscript describes the sequencing, assembly, and analysis of the genome of yellow catfish (Pelteobagrus fulvidraco). The authors utilized long fragment sequencing and Hi-C scaffolding to produce chromosome-level scaffolds, then short sequences to help validate long sequences and correct consensus errors. They provided analyses to estimate gene content. This genome will be useful for basic biological studies of the yellow catfish and also for use in agriculture. The comments below are intended to better clarify the information provided.<br><br>Estimated genome size was calculated using kmer-based calculation. This can be performed on unfiltered Illumina data, and kmers with low frequency are removed from the calculation. In their figure, this occurs at a frequency of about 15 - anything below that frequency is untrusted and is likely sequencing artifact. The authors should also make this calculation using other kmer lengths (at least 21-mers and 25-mers) to ensure this is a robust estimate.<br>Reply: Thanks a lot for the suggestion. We have used the method mentioned by the reviewer and re-analyzed the Illumina data with Kmer of 17, 21, 25 and 27. The estimated genome size ranged from 706 to 714 Mb. We have added the results into the revised manuscript in line 135 and Supplementary Table 1.<br><br>The assembly was polished using arrow and pilon (misspelled in line 7). The latest recommendation from the National Human Genome Research Institute is to use pilon to correct only indels because the short Illumina reads can be misaligned within repetitive regions and incorrectly polish the sequence.<br>Reply：We thanks a lot for the reviewer's reminding. The typo of pilon was correct in the line 156. We have tested the effects of the pilon on the base error correction. The

results confirmed that pilon could significantly reduce both substitution and InDel errors (data will be published in our following paper). Therefore, we applied pilon to correct both snp and InDel for the assembled genome in this work.

Was blood from the genome reference fish used for Hi-C analysis, or was this blood from a different animal?
Reply: We used the same individual for genome reference sequencing and Hi-C experiment. We have added the description in line 164 of the revised manuscript.

There is no information on the average contig length or range of lengths, or the number and distribution of gaps within each chromosomal scaffold. Although the contig N50 is 1.1 Mb, there are still 2,440 contigs in the assembly, which suggests there are many small contigs.
Reply: The reviewer's concern is very important. We have added a length distribution figure (Supplementary Figure 2) in the revised manuscript, showing the range of contig length. We also added a figure (Supplementary Figure 3) to show the length distribution of anchored and unanchored contigs, showing that the length of the unanchored contigs were obviously smaller than the anchored contigs. We therefore speculated that short lengths of unanchored contigs limited effective Hi-C reads mapping, leading to insufficient supporting evidence for their clustering, ordering and orientation on chromosomes. The gap distribution along chromosome was also shown in the Supplementary Figure 4. We found that gaps were enriched on two ends of chromosomes. Gap distribution of on chromosomes could be explained the distribution of repeat at chromosome terminals.

What is the final statistic on contig numbers, length, scaffolds, etc. for the submission?
Reply: We have added the Supplementary Table 2 in the revised manuscript according to the reviewer's suggestion.

How many contigs are there per chromosome? A simple table would suffice.
Reply: The supplementary table 2 were added in the revised manuscript to show the detailed contig information for chromosomes.

What was the average length of the 1,224 contigs that were removed during chromosomal scaffolding?
Reply：Using Hi-C data, we anchored 2,440 of 3,652 contigs into chromosomes. Those contigs (sequence number 1,212) were not removed, but was left in the final assembly as unanchored sequences. The average length of those 1,212 was 35.0 kb, which was significantly lower than that of the whole genome contigs (Supplementary Figure 3).

In Figure 4, the authors place seahorse phylogeny somewhere within teleost phylogeny. They should carefully examine their tree and compare it to previously published phylogenetic trees, with justifications when their results differ from the vast array of available phylogenies.
Reply：The reviewer's concern is very important. We have examined the phylogenetic results with the seahorse genome literature1, and found our result was consistent with the study. We thank the reviewer for the important reminding. We have added the reference in our revised manuscript in line 281.

There are no Figure Legends, and the information on the figures is insufficient. Figures should stand alone. For example, in Fig 2, what does the scale of 2-12 represent on the right? In Fig 3, which genomes are included in the black dots? In Supp Fig2, what do the colors represent?
Reply：We thank the reviewer for the reminding. We have added the detailed legends for figures. The color bar in Figure 2 illuminated the logarithm of the contact density from red (high) to white (low) in the plot. The statistics of 44 teleost genomes (43 public and the P. fulvidraco genome) were included in the Fig 3. We have added Supplementary Table 3 to include the statistics of genomes in Figure 3. In Supp Fig 2, the color represented the value of density. We have added the detailed legends for figures and tables in the revised manuscript.

Supplementary Figure 3 provides useful information and demonstrates the quality of the assembly. If Figures are limited, the authors may consider exchanging this with

Figure 3.
Reply： We thank the reviewer's constructive suggestion. We have added the Supplementary Figure 3 as Figure 4 in the revised manuscript.

Pdf page 8, Line 32 -The accuracy of 99.997%, as calculated by 21,143/780,000,000 bp, assumes complete homozygosity of the genome reference donor. Was this a homozygous fish? Otherwise, these SNPs could represent heterozygous loci within this fish or could represent assembly consensus artifact. This is also confounded with potential misalignment of Illumina reads in repetitive regions. Thus, an 'accuracy' estimate is complicated and hard to estimate.
Reply：The reviewer is correct that the "accuracy" of the genome assembly was complicated and hard to estimated. To avoid the mis-understanding, we have deleted the sentence of the genome accuracy from our manuscript.

Minor corrections:
Will the RRID citations be replaced with URLs?
Reply：We used the RRID for software used in this work because the GigaScience journal recommends the RRID. We have added a list of software and URLs at the end of the revised manuscript.

Pdf page 7, Line 16 - SDS molecules were quenched. Is "quenched" the correct term?
Reply: Thank for the reviewer's reminding. In this experimental step, Triton X-100 was used to quench the SDS to prevent it from denaturing enzymes in subsequent steps. We used the similar experimental steps and description as the following the reference2.

Pdf page 7, Line 39 - Please provide a reference for the 'previous study'.
Reply: Thanks for the reminding. We have added the citation in the revised manuscript (line 183).

Pdf page 8, Line 3 - do you mean 'contig number' instead of 'sequence number'?
Reply: We have corrected the sequencing number to contig number as reviewer's suggestion (line 204).

Pdf page 8, Line 23 - Which BUSCO database was used for this comparison?
Reply: The actinopterygii_odb9 database in BUSCO was used in our analysis. We have added the information in our revised manuscript in line 225.

Pdf page9, lines 13 and 31 - 'with a maximal e-value'
Reply：We have corrected the manuscript according to the reviewer's suggestion in line 258 and 268.

Reviewer #2: This manuscript describes the assembly of the yellow catfish genome, using state-of-the-art methodology. I have a few questions/comments on the text, methodology and results, that I would ask the authors to address:

Page 2, line 12: ' genome character evaluation', I assume this refers to nucleotide identity (using Illumina sequence) as contrasted with structural assembly (using PacBio data)? Also, what were the lengths of the Illumina reads?
Reply：Sorry for the confound to the reviewer. Here "genome character evaluation" means using NGS data to evaluated the genome size, the level of heterozygosity and repeat content in the genome. We have added the description to clarify in line 114-115. The length of Illumina reads was 150 bp.

Page 3, line 2: I am not familiar with the Genome Puzzle Master method. Perhaps you could describe the methodology is some detail. For example, what are its assumptions when merging assemblies? Do these fit two long-read assemblies as input data? How does the method end up with a much larger (730 Mbp) assembly than either of the inputs (both around 690 Mbp)?
Reply：Genome puzzle master (GPM) is a tool to build and edit pseudomolecules from fragmented sequences using sequence relationships.3 Since overlap information among contigs from two genomes can be used to guide the genome assembly, one important application of the GPM is to improve the genome assembly through

sequence-to-sequence alignments. Based on complements of two genomes, the contig could be elongated and the gaps are filled by sequences bridging two contigs. The method was used to improved the rice genome assemblies based on PacBio sequencing.4 We have added the more information and the reference for the application of GMP in line 146-151 of the revised manuscript.

Page 3, line 5: plion -> Pilon
Reply: Thanks for the reviewer for the reminding. We have correct the typo in line 156 of the revised manuscript.

Page 3, HiC description: This section is much more detailed than the others, perhaps streamline this a bit. In the methods, I actually miss the crosslinking step?
Reply：Thanks a lot for the reviewer. We have added the detailed steps for the crosslinking step for our Hi-C experiments in line 166-170 of the revised manuscript.

Page 3, lines 40/55: please cite the ' previous study'/'previous reports'
Reply：We have added the citation for the previous study to support the sentence in line 183.

Page 4, line 33: 'homologous SNP' -> homozygous SNP?
Reply: Thanks a lot for the reviewer. We have corrected the typo in the line 235 of the revised manuscript.

Figure 2: Please add a scale for the heatmap. Also, the assembly size used appears to be a 690 Mbp one instead of the final 730 Mbp assembly?
Reply：Thanks for the reviewer's reminding. We only illuminate the contact heatmap for sequences anchored in chromosomes, with a total length of 690 Mb. We have added the information in the legend of the figure.

Figure 3: I a quite sure there are more than five teleost assemblies with contig N50s over 100 kbp. The scaffold N50 scale is, for the interesting assemblies, less of a measure of assembly quality than an illustration of chromosome length.
Reply：We have included a supplementary table 3 for the fish species that we used in Figure 3. However, not every fish were assembled into chromosome level. Therefore, we only showed scaffold N50 in the Y axis.

General comment: as a major biological interest for the use of this genome assembly is the study of sex determination, and you used a female (XY) specimen, I assume you could already identify the two sex chromosomes in the assembly (using either coverage or heteryzygosity)?
Reply: We used XX female for genome assembly. The reviewer is correct. We have developed sex-specific markers in our previous studies5 and could help us to identified putative sex chromosome. The application of the genome on the sex-determination studies of the yellow catfish will be illuminated in our following reports.

Reviewer #3: In this manuscript Gong and colleagues reports the genome assembly of the yellow catfish (Pelteobagrus fulvidraco), an economically important freshwater fish manly farmed in China. This fish species exhibits a remarkable sex dimorphism on growth rate. Considering its economic relevance, the draft genome of the yellow catfish will be a valuable resource to facilitate future research aimed at improving relevant traits, and more generally at addressing ecological and evolutionary questions.

The authors used an adequate amount of sequence data coming from three different technologies (short reads, long reads and Hi-C), and this allowed to generate a robust chromosome-level genome. The workflow to assemble the genome sounds good and it is generally well described, even though some steps need to be better explained. Further, the authors annotated the genome using a combination of ab initio and homology-based methods that allowed them to identify a number of genes that is comparable to what has usually been found in other teleost fishes. Finally, they carried out some comparative genomics analyses including a bunch of other fish species in order to place the yellow catfish in well-defined phylogenetic context and to analyse the expansion/contraction of gene families in this lineage.

That said, I think that this manuscript needs some revision before to be considered for

Powered by Editorial Manager® and ProduXion Manager® from Aries Systems Corporation

publication in GigaScience. My main concern is about the language as the manuscript suffers from lack of clarity in several sections. Many sentences would benefit from being re-written and in general all the manuscript should be proofread before to be resubmitted to this or to any other journal.

Minor points:
Abstract, Finding:
- Change "The sequencing results were assembled…" to "The sequencing data were assembled…"
Reply: Thanks a lot for the reviewer's correction. We have revised the manuscript in line 39.

Introduction:
- The introduction is quite short. I would suggest the authors to expand the first section focusing on the species description, what are its distinctive traits, what type of studies have been done so far and to address which questions etc.
Reply: Thank a lot for the reviewer's constructive suggestion. We have expanded our introduction section for the description and recent research progresses of the yellow catfish in line 67-86 of the revised manuscript.

- As it appears here for the first time, please introduce "Hi-C" with its full name "Chromosome conformation capture"
Reply: Thanks for the reviewer's suggestion. We have added the introduction of Hi-C in the revised manuscript in line 160-165.

Sample and sequencing:
- "…platform according to the according…"
- PacBio flow cell should be "PacBio SMRT Cell"
Reply：We have revised the sentences in line 138 of the revised manuscript.

Genome quality evaluatuion:
- Which version of BUSCO did you use? And which database? The CVG (Core Vertebrate Genes) or the whole vertebrate gene set? Please include the number of genes that matched the database used, not only the percentage.
Reply：BUSCO v3.0 and actinopterygii_odb9 database was used for the genome evaluation. We have added the BUSCO version, database type and detailed output of the analysis in our revised manuscript (line 224-225).

- "Using the Illumina short…". Can the authors explain what they exactly did here?
Reply：The Illumina short reads were aligned to the reference genome, and the SNP loci were called through GATK pipeline. We have added the detailed method in the revised manuscript (line 233-234).

Conclusion:
- Here at the beginning of the paragraph, I would mention that you also used Illumina short reads.
Reply：Thanks for the reminding. We have added the Illumina short read in the revised manuscript in line 304.

Software version is missing several times along the text, and different styles are used to cite a software. Please revise and make it consistent.
Reply：We have added the software version information and revised the citation for the software.

Table 1:
- I would slightly change the first 2 columns of table 1:
Library type: "short reads", "long reads", "Hi-C"
Reply：We have revised the table information according to the reviewer's suggestion in Table 1.

Figure 1 is quite ugly. I suggest the authors to look for a better image or take a picture themselves.
Reply: We have replaced the Figure 1 in our revised manuscript. Thank the reviewer for the suggestion.

	Figure 3: The legend is not so informative. What are the black and the red dots? I know they indicate different fish species, but what are the criteria to make them red or black? Please add this info in the legend.<br>Reply: The red dots in Figure 3 represented the teleost genomes that totally assembled using long reads from the third-generation sequencing platform. We have added the detailed the legends for all figures and tables in the revised manuscript.<br><br>References<br>1Qiang, L. et al. The seahorse genome and the evolution of its specialized morphology. Nature 540, 395-399 (2016).<br>2Belton, J. M. et al. Hi-C: a comprehensive technique to capture the conformation of genomes. Methods 58, 268-276 (2012).<br>3Zhang, J. et al. in International Plant and Animal Genome Conference Xx.<br>4Zhang, J. et al. Extensive sequence divergence between the reference genomes of two elite indica rice varieties Zhenshan 97 and Minghui 63. Proc Natl Acad Sci U S A 113, E5163 (2016).<br>5Wang, D., Mao, H. L., Chen, H. X., Liu, H. Q. & Gui, J. F. Isolation of Y- and X-linked SCAR markers in yellow catfish and application in the production of all-male populations. Animal Genetics 40, 978-981 (2010).
**Additional Information:**	
**Question**	**Response**
Are you submitting this manuscript to a special series or article collection?	No
**Experimental design and statistics**<br><br>Full details of the experimental design and statistical methods used should be given in the Methods section, as detailed in our Minimum Standards Reporting Checklist. Information essential to interpreting the data presented should be made available in the figure legends.<br><br>Have you included all the information requested in your manuscript?	Yes
**Resources**<br><br>A description of all resources used, including antibodies, cell lines, animals and software tools, with enough information to allow them to be uniquely identified, should be included in the Methods section. Authors are strongly encouraged to cite Research Resource Identifiers (RRIDs) for antibodies, model organisms and tools, where possible.	Yes

Have you included the information requested as detailed in our Minimum Standards Reporting Checklist?	
**Availability of data and materials**<br><br>All datasets and code on which the conclusions of the paper rely must be either included in your submission or deposited in publicly available repositories (where available and ethically appropriate), referencing such data using a unique identifier in the references and in the “Availability of Data and Materials” section of your manuscript.<br><br>Have you have met the above requirement as detailed in our Minimum Standards Reporting Checklist?	Yes

Powered by Editorial Manager® and ProduXion Manager® from Aries Systems Corporation

Manuscript

Click here to view linked References

Click here to access/download;Manuscript;manuscript_r1_SCE_Revised.do

Chromosomal-level assembly of yellow catfish genome using third-generation DNA sequencing and Hi-C analysis

Gaorui Gong[1,#], Cheng Dan[1,#], Shijun Xiao[2,#], Wenjie Guo[1], Peipei Huang[3], Yang Xiong[1], Junjie Wu[1], Yan He[1], Jicheng Zhang[2], Xiaohui Li[1], Nansheng Chen[4,5], Jian-Fang Gui[1,3,]*, Jie Mei[1,]*

[1] College of Fisheries, Key Laboratory of Freshwater Animal Breeding, Ministry of Agriculture, Huazhong Agricultural University, Wuhan, China.

[2] Wuhan Frasergen Bioinformatics, East Lake High-Tech Zone, Wuhan, China.

[3] State Key Laboratory of Freshwater Ecology and Biotechnology, Institute of Hydrobiology, Chinese Academy of Sciences, University of the Chinese Academy of Sciences, Wuhan, China.

[4] Institute of Oceanology, Chinese Academy of Sciences, Qingdao, Shandong, China

[5] Department of Molecular Biology and Biochemistry, Simon Fraser University, Burnaby, Canada

[#] These authors contributed equally to this work.

***** Corresponding author. Tel: +86-27-87282113; Fax: +86-27-87282114.

Email address: jmei@mail.hzau.edu.cn (Dr. Jie Mei, ORCID: 0000-0001-5308-3864)

jfgui@ihb.ac.cn (Dr. Jian-Fang Gui)

Abstract

Background: The yellow catfish, *Pelteobagrus fulvidraco*, belonging to Siluriformes order, is an economically important freshwater aquaculture fish species in Asia, especially in Southern China. The aquaculture industry has recently been facing tremendous challenges in germplasm degeneration and poor diseases resistance. As the yellow catfish exhibits notable sex dimorphism in growth, with adult males about two to three fold bigger than females, how aquaculture industry takes advantage of such sex dimorphism is another challenge. To address these issues, a high-quality reference genome of the yellow catfish would be a very useful resource.

Finding: To construct a high-quality reference genome for the yellow catfish, we generated 51.2 Gb short reads and 38.9 Gb long reads using Illumina and PacBio sequencing platforms, respectively. The sequencing data were assembled into a 732.8 Mb genome assembly with a contig N50 length of 1.1 Mb. Additionally, we applied Hi-C technology to identify contacts among contigs, which were then used to assemble contigs into scaffolds, resulting in a genome assembly with 26 chromosomes, and a scaffold N50 length of 25.8 Mb. Using 24,552 protein-coding genes annotated in the yellow catfish genome, the phylogenetic relationships of the yellow catfish with other teleosts showed that yellow catfish separated from the common ancestor of channel catfish ~81.9 million years ago. 1,717 gene families were identified to be expanded in the yellow catfish and those gene families are mainly enriched in immune system, signal transduction, glycosphingolipid biosynthesis and fatty acid biosynthesis.

Conclusion: Taking advantage of Illumina, PacBio and Hi-C technologies, we constructed the first high-quality chromosomal-level genome assembly for the yellow catfish *P. fulvidraco*. The genomic resources generated in this work not only offer a valuable reference genome for functional genomics studies of yellow catfish to decipher the economic traits and sex determination, but also provide important chromosome information for genome comparisons in the wider evolutionary research community.

Key Words: yellow catfish, PacBio, Hi-C, genomics, chromosomal assembly

Data description

Introduction

The yellow catfish, *Pelteobagrus fulvidraco*, (Richardson, 1846; NCBI Taxonomy ID: 1234273; Fishbase ID: 28052) is a teleost fish belonging to the order Siluriformes (**Figure 1**), and is an economically important freshwater fish species in Asia.[1] In recent years, yellow catfish has become one of the most important aquaculture species in China with an increasing market value because of its high meat quality and lack of intermuscular bones besides the spine[2]. However, due to the ultra-intensive aquaculture and loss of genetic diversity, artificial breeding of yellow catfish is facing tremendous challenges such as germplasm degeneration and poor diseases resistance[3]. Meanwhile, as an XY sex-determining type fish species, yellow catfish is also an excellent model for studying sex determination and sexual size dimorphism in fish[4,5]. As female and male yellow catfish exhibit remarkable sex dimorphism in their growth rate, with adult yellow catfish males about two to three fold bigger than the females. In the last decade, sex-specific allele markers were developed and YY super-male fish were generated from gynogenesis of XY physiological female fish. Finally, XX male, XY female, YY super-male and females have been created and provide a unique model to study sex determination in fish species[1,6,7]. Recently, transgene and gene knockout technologies have been successively applied in yellow catfish to reveal the function of pfpdz1 gene, a novel PDZ domain-containing gene, in whose intron the sex-linked marker was located. The pfpdz1 gene plays an important role in male sex differentiation and maintenance in yellow catfish[8]. Taken together these features provide a platform for gene-editing methods to study gene function.

In spite of the importance of yellow catfish both in sex-determination research and in aquaculture, the genomic resources for the species are still limited. So far, only transcriptome, SSR and SNP data have been reported for yellow catfish[5], the genome sequence for this important species is still missing, hindering the genome-based functional gene identification controlling important economic traits and the application of genome-assisted breeding in yellow catfish. In this work, we combined genomic sequencing data from Illumina short reads and PacBio long reads to generate the first reference genome for yellow catfish, and applied Hi-C data to scaffold the genome sequences into the chromosomal level. The completeness and continuity of the genome were comparable with other model teleost species. We believe that the high-quality reference genome generated in this work will definitely facilitate research on population genetics and functional genes identification related to important economic traits and the sex determinant for yellow catfish, which will in turn accelerate the development of more efficient sex control techniques and improve the artificial breeding industry for this

economically important fish species.

Sample and sequencing

A XX genotype female yellow catfish (Figure 1), reared in the breeding center of Huazhong Agricultural University in Wuhan City, Hubei Province, was used for preparing DNA for sequencing. To obtain sufficient high-quality DNA molecules for the PacBio Sequel platform (Pacific Biosciences of California, Menlo Park, CA, USA), one yellow catfish was dissected and fresh muscle tissues were used for DNA extraction using the phenol/chloroform extraction method as in previous study[9]. The quality of the DNA was checked by agarose gel electrophoresis, and an excellent integrity of DNA molecules were observed. Other tissues, including ocular, skin, muscle, gonadal, intestinal, liver, kidney, blood, gall and air bladder tissues were snap frozen in liquid nitrogen for at least one hour and then stored at −80 °C.

The extracted DNA molecules were sequenced with both Illumina HiSeq X Ten platform (Illumina Inc., San Diego, CA, USA) and PacBio Sequel platforms. Short reads generated from the Illumina platform were used for the estimation of the genome size, the level of heterozygosity and repeat content of the genome, and long reads from the PacBio platform were used for genome assembly. To this end, one library with an insertion length of 250 bp was generated for the HiSeq X Ten platform and three 20 kb libraries were constructed for the PacBio platform according to the manufacturer’s protocols, resulting the generation of ~51.2 Gb short reads and ~38.9 Gb long reads, respectively. (**Table 1**) The polymerase and subreads N50 length reached 21.3 kb and 16.2 kb, providing ultra-long genomic sequences for the following assembly.

Genome features estimation from *K*mer method

The short-reads from Illumina platform were quality filtered by HTQC v1.92.3[10] using the following method. Firstly, the adaptors were removed from the sequencing reads. Second, read pairs were excluded if any one end has an average quality lower than 20. Third, ends of reads were trimmed if the average quality lower than 20 in the sliding window size of 5 bp. Finally, read pairs with any end was shorter than 75 bp were removed.

The quality filtered reads were used for genome size estimation. Using the *K*mer method described in previous method[11], we calculated and plot the 17-mer depth distribution in SI Figure 1. The formula $G = N_{17\text{-mer}} / D_{17\text{-mer}}$,where the $N_{17\text{-mer}}$ is the total number of 17-mers,and $D_{17\text{-mer}}$ denotes the peak frequency of 17-mers, were used to estimate the genome size of yellow catfish. As a result, we estimated a genome size of 714 Mb, as well as a heterozygosity rate of 0.45% and repeat ratio of 43.31%. To confirm the robustness of the genome size estimation, we performed additional analysis with

Kmer of 21, 25 and 27, and found the estimated genome size ranged from 706 to 718 Mb (**Supplementary Table 1**).

Genome assembly by third-generation long reads

With 6 SMRT cells in PacBio Sequel platform, we generated 38.9 Gb subreads by removing adaptor sequences within sequences. The mean and N50 length were 9.8 and 16.2 kb, respectively. The long subreads were used for genomic assembly of yellow catfish. Firstly, Falcon v0.3.0 package [12] with a parameter of length_cutoff as 10 kb and pr_length_cutoff as 8 kb was used. As a result, we obtained a 690 Mb genome with a contig N50 length of 193.1 kb. Secondly, canu v1.5[13] was employed separately for genome assembly with default parameters, leading to 688.6 Mb yellow catfish genome with contig N50 of 427.3 kb.

Although the size of genome assembly from both Falcon and canu was comparable with the estimation based on *K*mer method, the continuity of the genome need further improvement. Genome puzzle master (GPM)[14] is a tool to guide the genome assembly from fragmented sequences using overlap information among contigs from genomes.[14] Based on the complementarity of the two genomes, the contig could be merged and the gaps filled by sequences bridging the two contigs.[15] Taking advantage of the sequence complementation of the two assemblies from Falcon and canu, we therefore applied GPM[14] to merge long contigs using reliable overlaps between sequences. Finally, a ~730 Mb genome assembly of yellow catfish with 3,564 contigs and contig N50/L50 of 1.1 Mb/126 was constructed. The final genome sequences were then polished by arrow[16] using PacBio long reads and by pilon release 1.12 [17] using Illumina short reads to correct errors in base level. The length distribution for contigs in the final assembly is presented in **Supplementary Figure 2**.

In situ Hi-C library construction and chromosome assembly using Hi-C data

Hi-C is a technique allowing to unbiased identify chromatin interactions across the entire genome[18]. The technique was introduced in as a genome-wide version of 3C (Capturing chromosome conformation)[19], and was used as a powerful tool in the chromosome genome assembly of many projects in recent years[20]. In this work, Hi-C experiments and data analysis on blood sample was used for the chromosome assembly of the yellow catfish. Blood sample from the same yellow catfish for genomic DNA sequencing was used for library construction for Hi-C analysis. 0.1 ml blood were cross-linked for 10 min with 1% final concentration fresh formaldehyde and quenched with 0.2 M final concentration glycine for 5 min. The cross-linked cells were subsequently lysed in lysis bufer (10 mMTris-HCl (pH 8.0), 10 mM NaCl, 0.2% NP40, and complete protease inhibitors (Roche)). The extracted nuclei were re-suspended with 150 µl 0.1% SDS and incubated at 65°C for 10 min, then SDS molecules were quenched by adding 120 µl water

and 30 μl 10% Triton X-100, and incubated at 37 °C for 15 min. The DNA in the nuclei was digested by adding 30 μl 10x NEB buffer 2.1(50 mM NaCl, 10 mM Tris-HCl, 10 mM $MgCl_2$, 100 μg/ml BSA, pH 7.9) and 150U of MboI, and incubated at 37 °C overnight. On the next day, the MboI enzyme was inactivated at 65 °C for 20 min. Next, the cohesive ends were filled in by adding 1 μl of 10 mM dTTP, 1 μl of 10 mM dATP, 1 μl of 10 mM dGTP, 2 μl of 5 mM biotin-14-dCTP, 14 μl water and 4 μl (40 U) Klenow, and incubated at 37 °C for 2 h. Subsequently, 663 μl water,120 μl 10x blunt-end ligation buffer (300 mM Tris-HCl, 100 mM $MgCl_2$, 100 mM DTT, 1 mM ATP, pH 7.8), 100 μl 10% Triton X-100 and 20 U T4 DNA ligase were added to start proximity ligation. The ligation reaction was placed at 16 °C for 4 h. After ligation, the cross-linking was reversed by 200 μg/mL proteinase K (Thermo) at 65°C overnight. Subsequent chromatin DNA manipulations were performed as a similar method described in the previous study[19]. DNA purification was achieved through QIAamp DNA Mini Kits (Qiagen) according to manufacturers` instructions. Purified DNA was sheared to a length of ~400 bp. Point ligation junctions were pulled down by Dynabeads® MyOne™ Streptavidin C1 (Thermofisher) according to manufacturers` instructions. The Hi-C library for Illumina sequencing was prepared by NEBNext® Ultra™ II DNA library Prep Kit for Illumina (NEB) according to manufacturers` instructions. The final library was sequenced on the Illumina HiSeq X Ten platform (San Diego, CA, United States) with 150 PE mode.

487 million raw reads were generated from the Hi-C library and were mapped to the polished yellow catfish genome using Bowtie 1.2.2 (RRID:SCR_005476) [21] with the default parameters. The iterative method was used to increase the interactive Hi-C reads ratio [22]. Two ends of paired reads were mapped to the genome independently, but only the reads that two pairs were uniquely mapped to genome were used. Self-ligation, non-ligation and other invalid reads, such as StartNearRsite, PCR amplification, random break, LargeSmallFragments and ExtremeFragments, were filtered using the method and hiclib as described in previous reports[23]. The contact count among each contig were calculated and normalized by the restriction sites in sequences (**Figure 2**). We then successfully clustered 2,965 contigs into 26 groups with the agglomerative hierarchical clustering method in Lachesis[24], which was consistent with the previous karyotype analyses of *Pseudobagrus fulvidraco*[25]. Lachesis was further applied to order and orient the clustered contigs, and 2,440 contigs were reliably anchored on chromosomes, presenting 66.8% and 94.2% of the total genome by contig number and base count, respectively. Then, we applied juicebox[26] to correct the contig orientation and to remove suspicious fragments in contig to unanchored groups by visual inspection. Finally, we obtained the first chromosomal-level high-quality yellow catfish assembly with a contig N50 of 1.1 Mb and scaffold N50 of 25.8 Mb, providing solid genomic resource for the following population and functional analysis. (**Table 2**). We compared length distribution of

contig anchored and un-anchored on chromosomes (Supplementary Figure 3), and found that anchored contigs were significantly longer than those of unanchored contigs. We therefore speculated that short lengths of unanchored contigs limited effective Hi-C reads mapping, leading to insufficient supporting evidence for their clustering, ordering and orientation on chromosomes. The gap distribution on chromosomes are shown in Supplementary Figure 4. We found that gaps were mainly distributed at two ends of chromosomes, which could be explained by the repeat distribution at chromosome terminals. The length and the statistics of contigs and gaps of each chromosome were summarized in **Supplementary Table 2**.

Genome quality evaluation

First of all, we compared the genome assembly continuity of the yellow catfish genome to those of other teleost species. We found that both contig and scaffold N50 lengths of the yellow catfish reached considerable continuity (Figure 3), providing us a high-quality genome sequences for the following functional investigations. The assembled genome were also subjected to BUSCO v3.0[27] (RRID:SCR_015008, version 3.0) with the actinopterygii_odb9 database to evaluate the completeness of the genome. Among 4,584 total BUSCO groups searched, 4,179 and 92 BUSCO core genes were completed and partially identified, respectively, leading to a total of 91.2% BUSCO genes in the yellow catfish genome. After aligning short reads from Illumina platform to the genome, the insertion length distribution for sequencing library of 250 bp exhibited a single peak around the sequencing library length design (Supplementary Figure 5). Paired-end reads data were not used during the contig assembly, thus the high alignment ratio and single peak insertion length distribution demonstrated the high-quality of contig assembly for yellow catfish. Using the Illumina short read alignment to the reference genome of the yellow catfish by BWA 0.7.16 software (RRID:SCR_010910), we identified 21,143 homozygous SNP loci by GATK (RRID:SCR_001876) package[28].

Repeat and gene annotation

We first used Tandem Repeat Finder[29] to identify repetitive elements in yellow catfish genome. RepeatModeler (http://www.repeatmasker.org/RepeatModeler.html, RRID:SCR_015027) were used to detect transposable elements (TE) in the genome by a *de novo* manner. The *de novo* and known repeats library from Repbase[30] were then combined, and the TEs were detected by mapping sequences to the combined library in yellow catfish genome using the software RepeatMasker 4.0.7 (RRID:SCR_012954)[31].

For protein-coding gene annotation, *de novo*-, homology- and RNA-seq-based methods were used. Augustus (RRID:SCR_008417)[32] was used to predict coding genes in *de novo* prediction. For homology-based method, protein sequences of closely related fish species, including *Astyanax mexicanus*, *Danio rerio*, *Gadus morhua*, *Ictalurus*

punctatus, Oryzias latipes，Takifugu rubripes，Tetraodon nigroviridis and *Oreochromis niloticus* were downloaded from Ensembl[33] and were aligned against to the yellow catfish genome using TBLASTN (RRID:SCR_011822) software[34]. Short reads from RNA-Seq (SRR1845493) were also mapped upon the genome using TopHat v2.1.1 (RRID:SCR_013035) package[35], and the gene structure were formed using Cufflinks (RRID:SCR_014597)[36]. Finally, 24,552 consensus protein-coding genes were predicted in the yellow catfish genome by integrating all gene models by MAKER[37]. The gene number, gene length distribution, CDS length distribution, exon length distribution and intron length distribution were comparable with those in other teleost fish species (Figure 4).

Local BLASTX (RRID:SCR_001653) and BLASTN (RRID:SCR_001598) programs were used to search all predicted gene sequences to NCBI non-redundant protein (nr), non-redundant nucleotide (nt), Swissprot database with a maximal e-value of $1e^{-5}$ [38]. Gene ontology (GO)[39] and Kyoto Encyclopedia of Genes and Genomes (KEGG)[40] pathway annotation were also assigned to genes using the software Blast2GO[41].As a result, 24,552 genes were annotated to at least one database. (Table 3)

Gene family identification and Phylogenetic analysis of yellow catfish

To cluster families from protein-coding genes, proteins from the longest transcripts of each genes from yellow catfish and other fish species, including *Ictalurus punctatus*, *Clupeaharengus*, *Danio rerio*, *Takifugu rubripes*, *Hippocampus comes*, *Cynoglossus semilaevis*, *Oryzias latipes*, *Gadus morhua*, *Lepisosteus oculatus*, *Dicentrarchus labrax*, and *Gasterosteus aculeatus,* were extracted and aligned to each other using BLASTP (RRID:SCR_001010) programs[38] with a maximal e-value of $1e^{-5}$. OrthMCL[42] was used to cluster gene family using protein BLAST result. As a result, 19,846 gene families were constructed for fish species in this work and 3,088 families were identified as single-copy ortholog gene families.

To reveal phylogenetic relationships among yellow catfish and other fish species, the protein sequences of single-copy ortholog gene family were aligned with MUSCLE 3.8.31 (RRID:SCR_011812) program[43], and the corresponding Coding DNA Sequences (CDS) alignments were generated and concatenated with the guidance of protein alignment. PhyML v3.3 (RRID:SCR_014629)[44] were used to construct the phylogenetic tree for the super-alignment of nucleotide sequences using the JTT+G+F model. Using molecular clock data from the divergence time from the TimeTree database[45], the PAML v4.8 MCMCtree program[46] was employed to determine divergence times with the approximate likelihood calculation method. The phylogenetic relationship of other fish species was consistent with previous studies[47]. The phylogenetic analysis based on single-copy orthologs of yellow catfish with other teleosts studied in this work estimated that the yellow catfish speciated around 81.9 million years ago from their common ancestor of the

channel catfish (Figure 5). Given yellow catfish and channel catfish belong to family Bagridae and Ictaluridae respectively, the phylogenetic analysis showed that Bagridae and Ictaluridae were separated at a comparable time scale, however, determining the exact time estimation requires more Siluriformes genomes.

Gene family expansion and contraction analysis

According to divergence times and phylogenetic relationships, CAFE[48] was used to analyze gene family evolution and 1,717 gene families were significantly expanded in the yellow catfish ($P < 0.05$). The functional enrichment on GO and KEGG of those expanded gene families identified 350 and 42 significantly enriched (q-value < 0.05) GO terms (**Supplementary Table 3**) and pathways (**Supplementary Table 4**), respectively. The expanded gene families were mainly found on immune system pathways, especially on Hematopoietic cell lineage (q-value = 2.2e-17), Intestinal immune network for IgA production (q-value = 2.4e-17), Complement and coagulation cascades (q-value = 1.4e-15) and Antigen processing and presentation (q-value = 2.3e-9) on KEGG pathways, and Signal transduction pathways, including NF-kappa B signaling pathway (q-value = 5.4e-9), Rap1 signaling pathway (q-value = 1.9e-6) and PI3K-Akt signaling pathway (q-value = 2.3e-4). Meanwhile, 208 GO terms and 44 KEGG pathways, including endocrine system, signal transduction, xenobiotics biodegradation and metabolism, sensory system were enriched using significantly contracted gene families.

Conclusion

Combining Illumina and PacBio sequencing platforms with Hi-C technology, we reported the first high-quality chromosomal level genome assembly for the yellow catfish. The contig and scaffold N50 reached 1.1 and 25.8 Mb, respectively. 24,552 protein-coding genes were identified in the assembled yellow catfish, and 3,088 gene families were clustered for fish species in this work. The phylogenetic analysis of related species showed that yellow catfish diverged ~81.9 MYA from the common ancestor of the channel catfish. Expanded gene families were significantly enriched in several important biological pathways, mainly in immune system and signal transduction, and important functional gene in those pathways were identified for following studies. Given the economic importance of yellow catfish and the increasing research interests for the species, the genomic data in this work offered valuable resource for functional gene investigations of yellow catfish. Furthermore, the chromosomal assembly of yellow catfish also provides valuable data for evolutionary studies for the research community in general.

Availability of supporting data

The raw sequencing and physical mapping data are available from NCBI via the accession number of SRR7817079, SRR7817060 and SRR7818403 via the project PRJNA489116; as well as the National Omics Data Encyclopedia (NODE) (http://www.biosino.org/node/index) via the project ID OEP000129 (http://www.biosino.org/node/project/detail/OEP000129). The genome, annotation and intermediate files and results are also available via the *GigaScience* GigaDB repository[49]. All supplementary figures and tables are provided in Supplemental Table 1-3 and Supplementary Figure 1-5.

Software and URLs

Software	URLs
HTQC	https://sourceforge.net/projects/htqc/
Falcon	https://github.com/PacificBiosciences/FALCON/wiki/Manual
Canu	https://github.com/marbl/canu
GMP	https://github.com/Jianwei-Zhang/LIMS
Pilon	https://github.com/broadinstitute/pilon/
Bowtie	http://bowtie-bio.sourceforge.net/index.shtml
Hiclib	https://bitbucket.org/mirnylab/hiclib/src
Lachesis	https://github.com/shendurelab/LACHESIS
Juicebox	https://www.aidenlab.org/juicebox/
BUSCO	https://busco.ezlab.org/
BWA	http://bio-bwa.sourceforge.net/
GATK	https://software.broadinstitute.org/gatk/
RepeatModeler	http://www.repeatmasker.org/RepeatModeler.html
RepeatMasker	http://repeatmasker.org/
Augustus	https://ngs.csr.uky.edu/Augustus
Balst	https://blast.ncbi.nlm.nih.gov/Blast.cgi
TopHat	https://ccb.jhu.edu/software/tophat/index.shtml
Cufflinks	http://cole-trapnell-lab.github.io/cufflinks/
MAKER	http://www.yandell-lab.org/software/maker.html
Blast2GO	https://www.blast2go.com/
OrthMCL	https://github.com/apetkau/orthomcl-pipeline
MUSCLE	http://www.drive5.com/muscle/
PhyML	https://github.com/stephaneguindon/phyml
TimeTree	http://timetree.org/
PAML	http://abacus.gene.ucl.ac.uk/software/paml.html

Abbreviations

3C: Capturing Chromosome Conformation; bp: base-pair; BUSCO: Benchmarking Universal Single-Copy Orthologs; CDS: Coding DNA Sequences; Gb: Gigabase; GO: Gene Ontology; Kb: kilobase; KEGG: Kyoto Encyclopedia of Genes and Genomes; Mb: megabase; Mya: Million years ago; PE: paired-end; TE: Transposable Element.

Competing interests

The authors declare that they have no competing interests.

Funding

This work was supported by China Agriculture Research System (CARS-46) and the Fundamental Research Funds for the Central Universities (2662017PY013).

Author Contributions

Jie Mei, Jian-Fang Gui and Nansheng Chen conceived the study; Dan Chen, Jicheng Zhang, Wenjie Guo and Peipei Huang collected the samples and performed sequencing and Hi-C experiments; Shijun Xiao, Gaorui Gong and Yan He estimated the genome size and assembled the genome; Shijun Xiao, Gaorui Gong and Xiaohui Li assessed the assembly quality; Gaorui Gong, Shijun Xiao, Yang Xiong and Junjie Wu carried out the genome annotation and functional genomic analysis, Jie Mei, Nansheng Chen, Shijun Xiao, Gaorui Gong and Jian-Fang Gui wrote the manuscript. And all authors read, edited, and approved the final manuscript.

References

1 Liu, H. *et al.* Genetic manipulation of sex ratio for the large-scale breeding of YY super-male and XY all-male yellow catfish (*Pelteobagrus fulvidraco* (Richardson)). *Marine Biotechnology* **15**, 321-328 (2013).

2 Zhang, J. *et al.* Characterization and development of EST-SSR markers derived from transcriptome of yellow catfish. *Molecules* **19**, 16402-16415 (2014).

3 Liu, F. *et al.* Effects of astaxanthin and emodin on the growth, stress resistance and disease resistance of yellow catfish (*Pelteobagrus fulvidraco*). *Fish & Shellfish Immunology* **51**, 125 (2016).

4 Jie, M. & Gui, J. F. Genetic basis and biotechnological manipulation of sexual dimorphism and sex determination in fish. *Science China Life Sciences* **58**, 124 (2015).

5 Chen, X. *et al.* A comprehensive transcriptome provides candidate genes for sex determination/differentiation and SSR/SNP markers in yellow catfish. *Marine Biotechnology* **17**, 190-198 (2015).

6 Dan, C., Mei, J., Wang, D. & Gui, J. F. Genetic Differentiation and Efficient Sex-specific Marker Development of a Pair of Y- and X-linked Markers in Yellow Catfish. *International Journal of Biological Sciences* **9**, 1043-1049 (2013).

7 Tian-Yi YANG, Y. X., Cheng DAN, Wen-Jie Guo, Han-Qin LIU, Jian-Fang GUI, Jie MEI. . Production of XX male yellow catfish by sex-reversal technology. *Acta Hydrobiologica Sinica* **42**, 871–878 (2018).

8 Dan, C., Lin, Q., Gong, G., et al. A novel PDZ domain-containing gene is essential for male sex differentiation and maintenance in yellow catfish (*Pelteobagrus fulvidraco*). *Science Bulletin* (2018). doi: 10.1016/j.scib.2018.08.012

9 Xiao, S. *et al.* Whole-genome single-nucleotide polymorphism (SNP) marker discovery and association analysis with the eicosapentaenoic acid (EPA) and docosahexaenoic acid (DHA) content in *Larimichthys crocea*. *Peerj* **4**, e2664 (2016).

10 Yang, X. *et al.* HTQC: a fast quality control toolkit for Illumina sequencing data. *Bmc Bioinformatics* **14**, 1-4 (2013).

11 Xu, P. *et al.* Genome sequence and genetic diversity of the common carp, *Cyprinus carpio*. *Nature Genetics* **46**, 1212-1219 (2014).

12 Chin, C. S. *et al.* Phased Diploid Genome Assembly with Single Molecule Real-Time Sequencing. *Nature Methods* **13**, 1050 (2016).

13 Koren, S. *et al.* Canu: scalable and accurate long-read assembly via adaptive k-mer weighting and repeat separation. *Genome Research* **27**, 722 (2017).

14 Zhang, J. *et al.* Genome puzzle master (GPM): an integrated pipeline for building and editing pseudomolecules from fragmented sequences. *Bioinformatics* **32**, 3058-3064, doi:10.1093/bioinformatics/btw370 (2016).

15 Zhang, J. *et al.* Extensive sequence divergence between the reference genomes of two elite indica rice varieties Zhenshan 97 and Minghui 63. *Proc Natl Acad Sci U S A* **113**, E5163 (2016).

16 Chin, C. S. *et al.* Nonhybrid, finished microbial genome assemblies from long-read SMRT sequencing data. *Nature Methods* **10**, 563 (2013).

17 Walker, B. J. *et al.* Pilon: An Integrated Tool for Comprehensive Microbial Variant Detection and Genome Assembly Improvement. *Plos One* **9**, e112963 (2014).

18 Lieberman-Aiden, E. & Dekker, J. Comprehensive Mapping of Long-Range Interactions Reveals Folding Principles of the Human Genome. *Science* **326**, 289 (2009).

19 Belaghzal, H., Dekker, J. & Gibcus, J. H. HI-C 2.0: An optimized hi-c procedure for high-resolution genome-wide mapping of chromosome conformation. *Methods* **123**, 56-65 (2017).

20 Dudchenko, O. *et al.* De novo assembly of the Aedes aegypti genome using Hi-C yields chromosome-length scaffolds. *Science* **356**, 92 (2017).

21 Langmead, B., Trapnell, C., Pop, M. & Salzberg, S. L. Ultrafast and memory-efficient alignment of short DNA sequences to the human genome. *Genome Biology* **10**, R25 (2009).

22 Nicolas, S. *et al.* HiC-Pro: an optimized and flexible pipeline for Hi-C data processing. *Genome*

Biology **16**, 259 (2015).

23 Xie, T., Yang, Q. Y., Wang, X. T., Mclysaght, A. & Zhang, H. Y. Spatial Colocalization of Human Ohnolog Pairs Acts to Maintain Dosage-Balance. *Molecular Biology & Evolution* **33**, 2368-2375 (2016).

24 Burton, J. N. *et al.* Chromosome-scale scaffolding of de novo genome assemblies based on chromatin interactions. *Nature Biotechnology* **31**, 1119-1125 (2013).

25 Shu-qun, X. Karyotype analyses of *Pseudobagrus fulvidraco*. *Chinese Journal of Fisheries* (2006).

26 Dudchenko, O. *et al.* The Juicebox Assembly Tools module facilitates de novo assembly of mammalian genomes with chromosome-length scaffolds for under $1000. (2018). bioRxiv 254797; doi: https://doi.org/10.1101/254797

27 Simão, F. A., Waterhouse, R. M., Ioannidis, P., Kriventseva, E. V. & Zdobnov, E. M. BUSCO: assessing genome assembly and annotation completeness with single-copy orthologs. *Bioinformatics* **31**, 3210 (2015).

28 Mckenna, A. *et al.* The Genome Analysis Toolkit: A MapReduce framework for analyzing next-generation DNA sequencing data. *Genome Research* **20**, 1297-1303 (2010).

29 Benson, G. Tandem repeats finder: a program to analyze DNA sequences. *Nucleic Acids Research* **27**, 573 (1999).

30 Bao, W., Kojima, K. K. & Kohany, O. Repbase Update, a database of repetitive elements in eukaryotic genomes. *Mobile Dna* **6**, 11 (2015).

31 Chen, N. Using RepeatMasker to identify repetitive elements in genomic sequences. *Current Protocols in Bioinformatics* **Chapter 4**, Unit 4.10 (2004).

32 Stanke, M. *et al.* AUGUSTUS: ab initio prediction of alternative transcripts. *Nucleic Acids Research* **34**, 435-439 (2006).

33 Flicek, P. *et al.* Ensembl 2014. *Nucleic Acids Research* **42**, D749-D755 (2014).

34 Gertz, E. M. *et al.* Composition-based statistics and translated nucleotide searches: Improving the TBLASTN module of BLAST. *Bmc Biology* **4**, 41 (2006).

35 Trapnell, C., Pachter, L. & Salzberg, S. L. TopHat: discovering splice junctions with RNA-Seq. *Bioinformatics* **25**, 1105-1111 (2009).

36 Ghosh, S. & Chan, C. K. K. Analysis of RNA-Seq Data Using TopHat and Cufflinks. *Methods in Molecular Biology* **1374**, 339 (2016).

37 Campbell, M. S., Holt, C., Moore, B. & Yandell, M. Genome Annotation and Curation Using MAKER and MAKER-P. *Current Protocols in Bioinformatics* **48**, 4.11.11 (2014).

38 Altschul, S. F., Gish, W., Miller, W., Myers, E. W. & Lipman, D. J. Basic local alignment search tool. *Journal of Molecular Biology* **215**, 403-410 (1990).

39 Harris, M. A. *et al.* The Gene Ontology (GO) database and informatics resource. *Nucleic Acids Research* **32**, D258-261, doi:10.1093/nar/gkh036 (2004).

40 Ogata, H. *et al.* KEGG: Kyoto Encyclopedia of Genes and Genomes. *Nucleic Acids Research* **27**, 29-34 (2000).

41 Conesa, A. *et al.* Blast2GO: a universal tool for annotation, visualization and analysis in functional genomics research. *Bioinformatics* **21**, 3674 (2005).

42 Li, L., Stoeckert, C. J. & Roos, D. S. OrthoMCL: Identification of Ortholog Groups for Eukaryotic Genomes. *Genome Research* **13**, 2178-2189 (2003).

43 Thompson, J. D., Gibson, T. J. & Higgins, D. G. *Multiple Sequence Alignment Using ClustalW*

and ClustalX. (John Wiley & Sons, Inc., 2002).

44 Guindon, S., Dufayard, J. F., Hordijk, W., Lefort, V. & Gascuel, O. PhyML: Fast and Accurate Phylogeny Reconstruction by Maximum Likelihood. **9**, 384-385 (2009).

45 Hedges, S. B., Dudley, J. & Kumar, S. TimeTree: a public knowledge-base of divergence times among organisms. *Bioinformatics* **22**, 2971-2972 (2006).

46 Yang, Z. PAML: a program package for phylogenetic analysis by maximum likelihood. *Computer Applications in Bioscience* **13**, 555-556 (1997).

47 Liu, Z. *et al.* The channel catfish genome sequence provides insights into the evolution of scale formation in teleosts. *Nature Communications* **7**, 11757 (2016).

48 De Bie, T., Cristianini, N., Demuth, J. P. & Hahn, M. W. CAFE: a computational tool for the study of gene family evolution. *Bioinformatics* **22**, 1269-1271 (2006).

49. Gong, G., Dan, C., Xiao, S. et al., Supporting data for " Chromosomal-level assembly of yellow catfish genome using third-generation DNA sequencing and Hi-C analysis". GigaScience Database, 2018. http://dx.doi.org/10.5524/100506

Tables and Figures

Tables

Table 1. Sequencing data generated for yellow catfish genome assembly and annotation. Note that paired-end 150 bp reads was generated from the Illumina HiSeq X Ten platform.

Library type	Platform	Library size (bp)	Data size (Gb)	Application
**Short reads**	HiSeq X Ten	250	51.2	genome survey and genomic base correction
**Long reads**	PacBio SEQUEL	20,000	38.9	genome assembly
**Hi-C**	HiSeq X Ten	250	146.1	chromosome construction

Table 2. Statistics for genome assembly of yellow catfish. Note that contigs were analyzed after the scaffolding based on Hi-C data.

Sample ID	Length		Number	
	Contig**(bp)	Scaffold(bp)	Contig**	Scaffold
**Total**	731,603,425	732,815,925	3,652	1,227
**Max**	11,531,338	55,095,979	-	-
**N50**	1,111,198	25,785,924	126	11
**N60**	643,552	24,806,204	212	14

**N70**	333,994	22,397,207	373	17
**N80**	128,419	21,591,549	742	21
**N90**	59,682	16,750,011	1,634	25

Table 3. Statistics for genome annotation of yellow catfish. Note that the e-value threshold of the 1e-5 was applied during the homolog searching for the functional annotation.

Database	Number	Percent
InterPro	20,178	82.18
GO	14,936	60.83
KEGG ALL	24,025	97.85
KEGG KO	13,951	56.82
Swissprot	20,875	85.02
TrEMBL	24,093	98.13
NR	24,308	99.01
Total	24,552	

Figures

Figure 1. Picture of a yellow catfish, *Pelteobagrus fulvidraco*. The fish was collected from the breeding center of Huazhong Agricultural University in Wuhan City, Hubei Province, China.

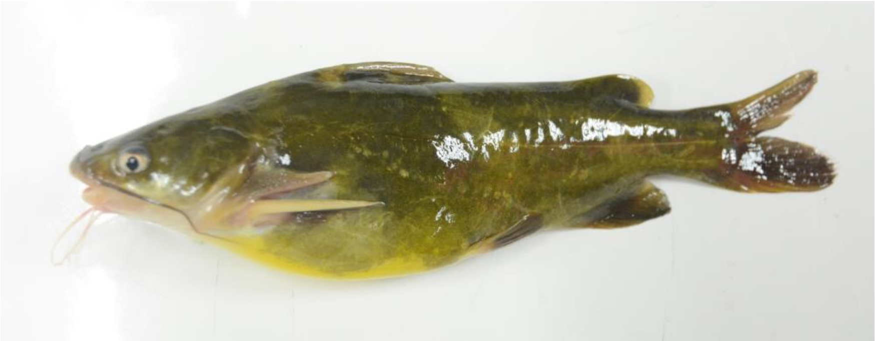

Figure 2. Yellow catfish genome contig contact matrix using Hi-C data. The color bar illuminated the logarithm of the contact density from red (high) to white (low) in the plot. Note that only sequences anchored on chromosomes were shown in the plot.

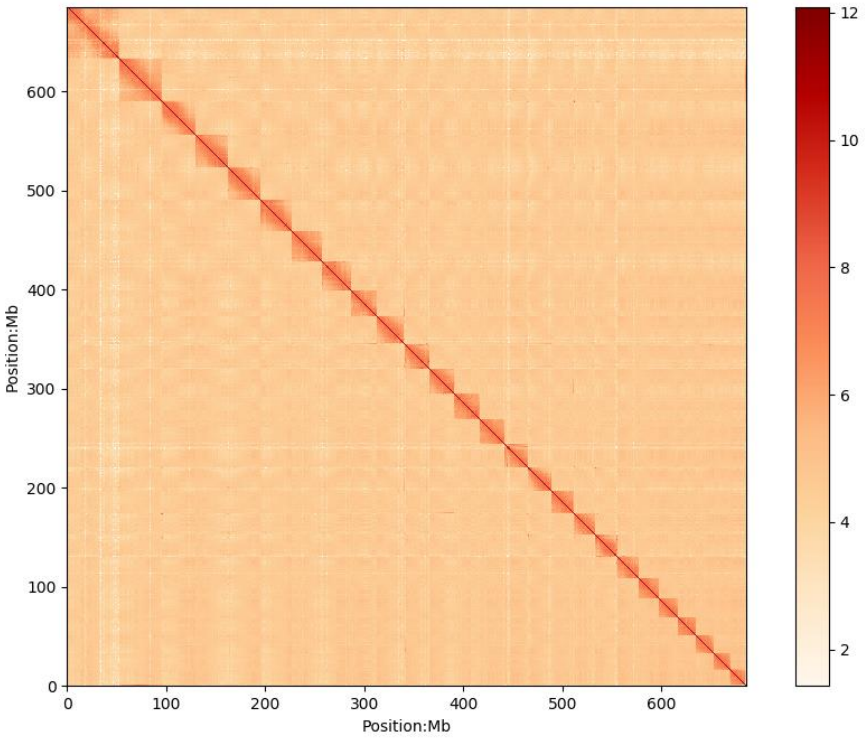

Figure 3. Genome assembly comparison of yellow catfish with other public teleost genomes. X and Y axis representing the contig and scaffold N50's, respectively. The genomes sequenced with third generation sequencing were highlighted in red.

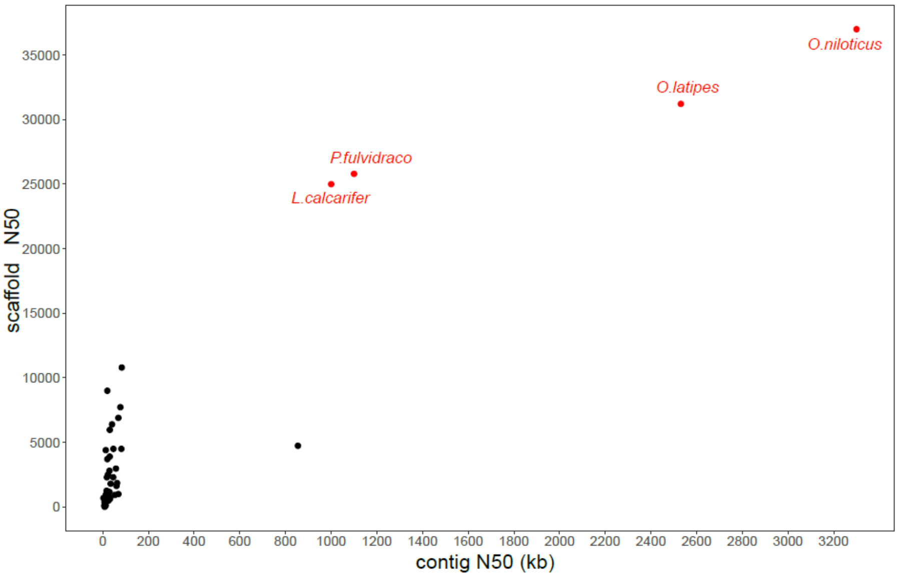

Figure 4. Length distribution comparison on total gene, CDS, exon and intron of annotated gene models of the yellow catfish with other closely related teleost fish species. Length distribution of total gene (A), CDS (B), exon (C) and intron (D) were compared to *P. fulvidraco, D. rerio, G. aculeatus, O. latipes, I. punctatus* and *T. rubripes*.

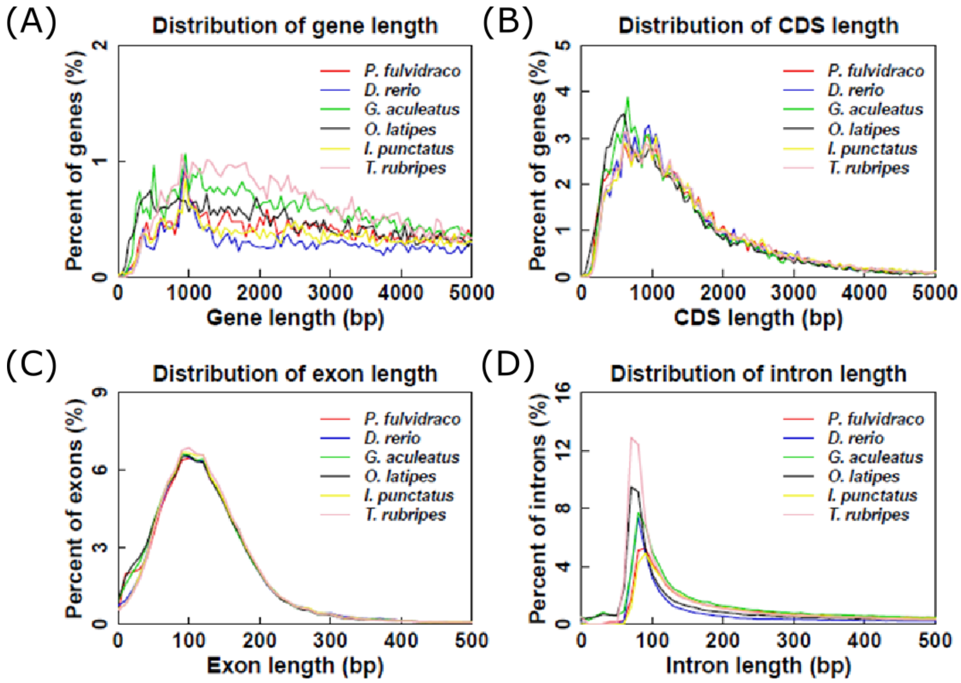

Figure 5. Phylogenetic analysis of the yellow catfish with other teleost species. The estimated species divergence time (MYA) and the 95% confidential intervals were labeled at each branch site. The divergence used for time recalibration was illuminated as red dots in the

tree. The fish (*I. punctatus* and *P. fulvidraco*) from the order Siluriformes were highlighted by pink shading.

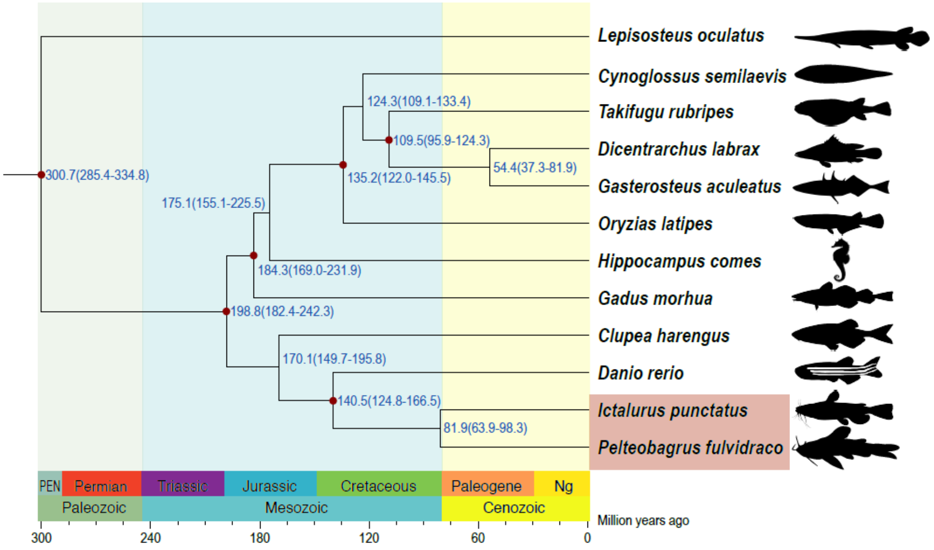

Click here to access/download
Supplementary Material
Supplementary information_R1.docx
