# Supplementary material for: Chromosomal-level assembly of yellow catfish genome using third-generation DNA sequencing and Hi-C analysis
Source: Gigascience. 2018 Sep 20;7(11):giy120. doi: 10.1093/gigascience/giy120 (PMC6228179; doi:10.1093/gigascience/giy120)

Supplementary Information for:

Chromosomal-level assembly of yellow catfish genome using third-generation DNA sequencing and Hi-C analysis

Gaorui Gong^1,#^, Cheng Dan^1,#^, Shijun Xiao^2,#^, Wenjie Guo^1^, Peipei Huang^3^, Yang Xiong^1^, Junjie Wu^1^, Yan He^1^, Jicheng Zhang^2^, Xiaohui Li^1^, Nansheng Chen^4,5^, Jian-Fang Gui^1,3,^*****, Jie Mei^1,^*****

^1^ College of Fisheries, Key Laboratory of Freshwater Animal Breeding, Ministry of Agriculture, Huazhong Agricultural University, Wuhan, China.

^2^ Wuhan Frasergen Bioinformatics, East Lake High-Tech Zone, Wuhan, China.

^3^ State Key Laboratory of Freshwater Ecology and Biotechnology, Institute of Hydrobiology, Chinese Academy of Sciences, University of the Chinese Academy of Sciences, Wuhan, China.

^4^ Institute of Oceanology, Chinese Academy of Sciences, Qingdao, Shandong, China

^5^ Department of Molecular Biology and Biochemistry, Simon Fraser University, Burnaby, Canada

^#^ These authors contributed equally to this work.

***** Corresponding author. Tel: +86-27-87282113; Fax: +86-27-87282114.

*Email address*: [jmei@mail.hzau.edu.cn](mailto:jmei@mail.hzau.edu.cn) (Dr. Jie Mei)

[jfgui@ihb.ac.cn](mailto:jfgui@ihb.ac.cn) (Dr. Jian-Fang Gui)

[chenn@sfu.ca](mailto:chenn@sfu.ca) (Dr. Nansheng Chen)

**Supplementary Table 1: Genome size estimation with Kmer distribution analysis using Kmer’s of 17, 21, 25 and 27.**

| Kmer size | 17 | 21 | 25 | 27 |
| --- | --- | --- | --- | --- |
| Genome Size (Mb) | 714.6 | 718.0 | 702.2 | 706.8 |

**Supplementary Table 2: the length and statistics of contigs and gaps for chromosomes.**

| Chromosome | Length  (Mb) | Contig number | N base (kb) | Gap number | Max contig length (Mb) | Min contig length (Mb) | Contig n50 (Mb) |
| --- | --- | --- | --- | --- | --- | --- | --- |
| chr1 | 55.10 | 220 | 109.5 | 219 | 5.62 | 0.03 | 0.82 |
| chr2 | 42.45 | 93 | 46 | 92 | 10.57 | 0.03 | 4.19 |
| chr3 | 34.63 | 127 | 63 | 126 | 7.10 | 0.01 | 1.08 |
| chr4 | 33.05 | 88 | 43.5 | 87 | 5.06 | 0.02 | 3.33 |
| chr5 | 32.96 | 113 | 56 | 112 | 7.63 | 0.03 | 1.18 |
| chr6 | 31.58 | 113 | 56 | 112 | 4.98 | 0.02 | 1.49 |
| chr7 | 30.84 | 134 | 66.5 | 133 | 2.35 | 0.01 | 0.59 |
| chr8 | 28.66 | 100 | 49.5 | 99 | 8.65 | 0.03 | 1.34 |
| chr9 | 27.84 | 108 | 53.5 | 107 | 9.99 | 0.02 | 0.98 |
| chr10 | 27.57 | 110 | 54.5 | 109 | 4.99 | 0.03 | 1.00 |
| chr11 | 25.79 | 100 | 49.5 | 99 | 9.28 | 0.03 | 3.98 |
| chr12 | 25.50 | 92 | 45.5 | 91 | 8.86 | 0.01 | 0.94 |
| chr13 | 25.25 | 61 | 30 | 60 | 8.31 | 0.03 | 2.41 |
| chr14 | 24.81 | 79 | 39 | 78 | 5.20 | 0.02 | 4.00 |
| chr15 | 24.20 | 92 | 45.5 | 91 | 5.96 | 0.02 | 1.12 |
| chr16 | 23.30 | 84 | 41.5 | 83 | 3.52 | 0.04 | 1.54 |
| chr17 | 22.40 | 56 | 27.5 | 55 | 9.18 | 0.02 | 3.02 |
| chr18 | 21.99 | 114 | 56.5 | 113 | 2.27 | 0.02 | 0.52 |
| chr19 | 21.95 | 98 | 48.5 | 97 | 2.77 | 0.03 | 0.98 |
| chr20 | 21.63 | 71 | 35 | 70 | 4.27 | 0.02 | 1.57 |
| chr21 | 21.59 | 61 | 30 | 60 | 7.79 | 0.02 | 1.32 |
| chr22 | 19.52 | 43 | 21 | 42 | 11.53 | 0.02 | 11.53 |
| chr23 | 18.22 | 86 | 42.5 | 85 | 2.00 | 0.02 | 0.49 |
| chr24 | 17.89 | 67 | 33 | 66 | 4.74 | 0.03 | 1.21 |
| chr25 | 16.75 | 63 | 31 | 62 | 1.86 | 0.03 | 0.89 |
| chr26 | 14.97 | 67 | 33 | 66 | 1.89 | 0.03 | 0.81 |
| unanchored | 42.38 | 1212 |  |  | 0.44 | 0.00 | 0.04 |

**Supplementary Table 3: the length statistics for public teleost reference genomes in Figure 3.**

| species | contig N50 (kb) | scaffold N50 (kb) |
| --- | --- | --- |
| *G.morhua* | 2.8 | 688 |
| *A.rostrata* | 7.397 | 86.641 |
| *T.orientalis* | 7.5 | 136 |
| *O.mykiss* | 7.7 | 383.6 |
| *S.histophorus* | 8.41 | 14.33 |
| *N.furzeri* | 9.3 | 247 |
| *N.coriiceps* | 11.6 | 219.1 |
| *L.chalumnae* | 12.7 | 924 |
| *E.electricus* | 12.7 | 104.2 |
| *N.brichardi/pulcher* | 13.2 | 4,400 |
| *T.nigroviridis* | 16 | 984 |
| *S.anshuiensis* | 16.708 | 1,251 |
| *S.rhinocerous* | 17.658 | 894 |
| *C.idellus(male)* | 18.2 | 2,300 |
| *M.zebra* | 20 | 3,700 |
| *M.mola* | 20 | 9,000 |
| *A.burtoni* | 21.9 | 1,200 |
| *X.maculatus* | 22 | 1,100 |
| *P.nyererei* | 22.6 | 2,500 |
| *L.crocea* | 25.7 | 499 |
| *C.semilaevis* | 26.5 | 867 |
| *O.niloticus* | 29.3 | 2,800 |
| *S.grahami* | 29.335 | 1,155 |
| *P.olivaceus* | 30.5 | 3,900 |
| *S.formosus(Golden)* | 30.73 | 5,970 |
| *S.scovelli* | 32.24 | 640.41 |
| *H.comes* | 34.7 | 1,800 |
| *C.idellus(female)* | 40.7 | 6,400 |
| *B.floridae* | 46 | 2,300 |
| *C.milii* | 46.6 | 4,500 |
| *T.rubripes* | 52.883 | 928.938 |
| *S.salar* | 57.6 | 2,970 |
| *S.formosus(Red)* | 60.19 | 1,630 |
| *S.formosus(Green)* | 62.8 | 1,850 |
| *L.oculatus* | 68.3 | 6,900 |
| *C.carpio* | 68.4 | 1,000 |
| *I.punctatus* | 77.2 | 7,726.8 |
| *C.argus* | 81.4 | 4,500 |
| *G.aculeatus* | 83.2 | 10,800 |
| *D.rerio* | 854.399 | 4,737.936 |
| *L.calcarifer* | 1,000 | 25,000 |
| *P.fulvifraco* | 1,100 | 25,800 |
| *O.latipes* | 2,530.934 | 31,218.53 |
| *O.niloticus* | 3,300 | 37,000 |

**Supplementary Figure 1: depth distribution of 17-mer generated from NGS sequencing data.**


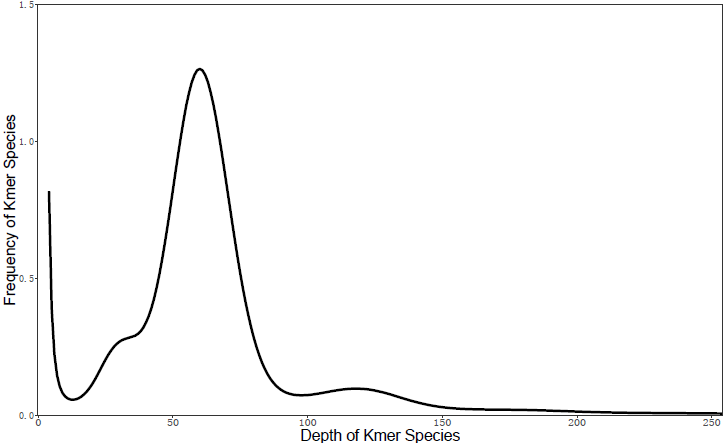


**Supplementary Figure 2: contig length distribution for the assembled genome.** Note that the red bar represents all contigs longer than 2 Mb.


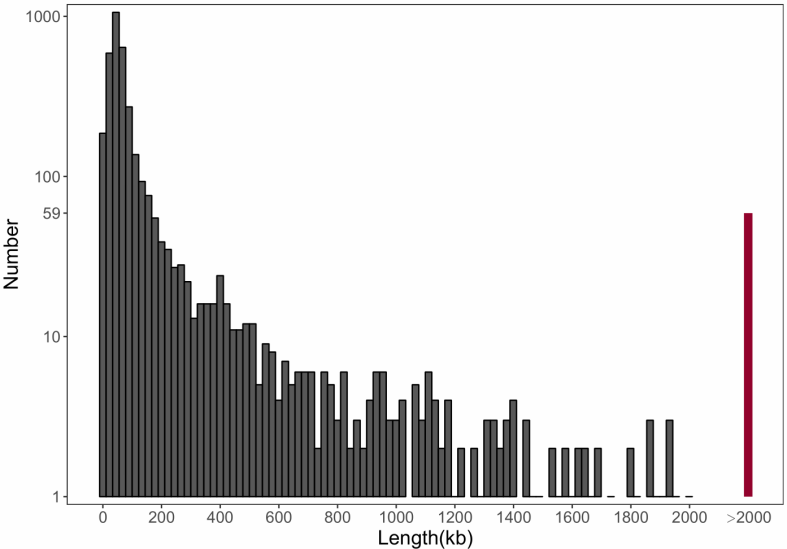


**Supplementary Figure 3: contig length distribution for contigs anchored (red) and un-anchored (blue) on chromosomes.** Note that contigs anchored on chromosomes were significantly higher value than those of unanchored contigs.


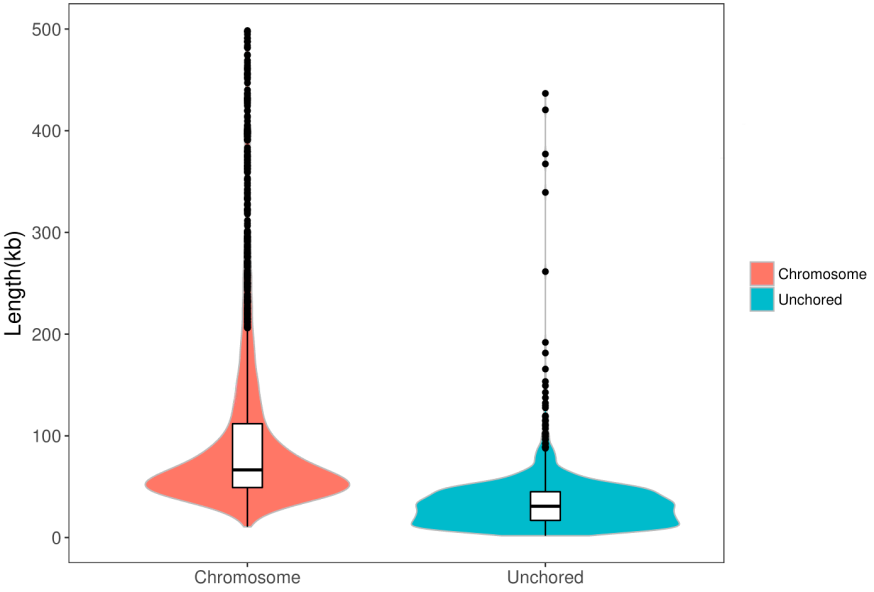


**Supplementary Figure 4: The distribution of gaps on chromosomes.** The black lines on each chromosome represent sequence gaps. Note that gaps were mainly distributed on two ends of each chromosome.


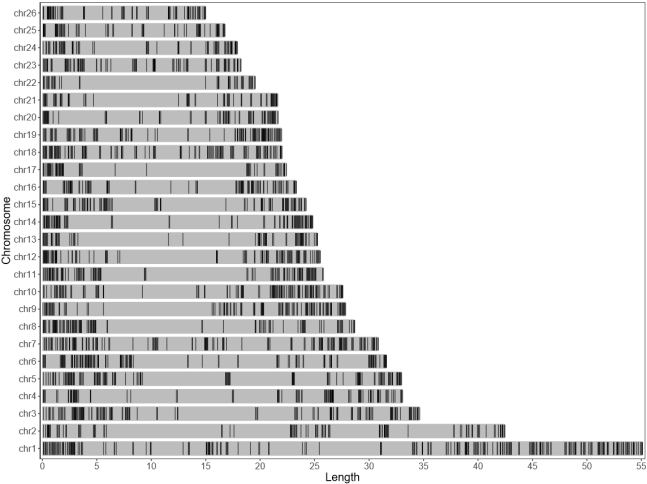


**Supplementary Figure 5: insertion length distribution of paired-end NGS sequencing data.** The color represents the percentage of reads.


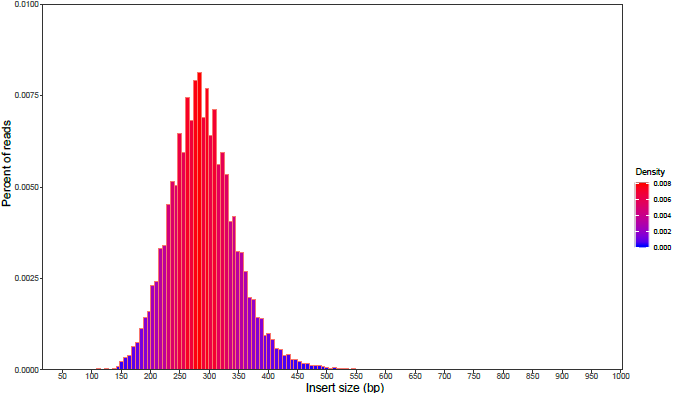

Supplement: Supplemental File [file giy120_supplemental_file.docx]
