# Supplementary material for: Chromosomal-level assembly of yellow catfish genome using third-generation DNA sequencing and Hi-C analysis
Source: Gigascience. 2018 Sep 20;7(11):giy120. doi: 10.1093/gigascience/giy120 (PMC6228179; doi:10.1093/gigascience/giy120)
Supplement: Reviewer_1_Report_(Original_Submission) -- Geoffrey Waldbieser — 7/23/2018 Reviewed [file giy120_reviewer_1_report_(original_submission).pdf]

Reviewer Report

Title: **Chromosomal-level assembly of yellow catfish genome using third-generation DNA sequencing and Hi-C analysis**

Version: **Original Submission** **Date:** 7/23/2018

Reviewer name: **Geoffrey Waldbieser**

Reviewer Comments to Author:

This manuscript describes the sequencing, assembly, and analysis of the genome of yellow catfish (Pelteobagrus fulvidraco). The authors utilized long fragment sequencing and Hi-C scaffolding to produce chromosome-level scaffolds, then short sequences to help validate long sequences and correct consensus errors. They provided analyses to estimate gene content. This genome will be useful for basic biological studies of the yellow catfish and also for use in agriculture. The comments below are intended to better clarify the information provided.

Estimated genome size was calculated using kmer-based calculation. This can be performed on unfiltered Illumina data, and kmers with low frequency are removed from the calculation. In their figure, this occurs at a frequency of about 15 - anything below that frequency is untrusted and is likely sequencing artifact. The authors should also make this calculation using other kmer lengths (at least 21-mers and 25-mers) to ensure this is a robust estimate.

The assembly was polished using arrow and pilon (misspelled in line 7). The latest recommendation from the National Human Genome Research Institute is to use pilon to correct only indels because the short Illumina reads can be misaligned within repetitive regions and incorrectly polish the sequence.

Was blood from the genome reference fish used for Hi-C analysis, or was this blood from a different animal?

There is no information on the average contig length or range of lengths, or the number and distribution of gaps within each chromosomal scaffold. Although the contig N50 is 1.1 Mb, there are still 2,440 contigs in the assembly, which suggests there are many small contigs.

What is the final statistic on contig numbers, length, scaffolds, etc. for the submission?
How many contigs are there per chromosome? A simple table would suffice.
What was the average length of the 1,224 contigs that were removed during chromosomal scaffolding?

In Figure 4, the authors place seahorse phylogeny somewhere within teleost phylogeny. They should carefully examine their tree and compare it to previously published phylogenetic trees, with justifications when their results differ from the vast array of available phylogenies.

There are no Figure Legends, and the information on the figures is insufficient. Figures should stand alone. For example, in Fig 2, what does the scale of 2-12 represent on the right? In Fig 3, which genomes are included in the black dots? In Supp Fig2, what do the colors represent?

Supplementary Figure 3 provides useful information and demonstrates the quality of the assembly. If Figures are limited, the authors may consider exchanging this with Figure 3.
Pdf page 8, Line 32 -The accuracy of 99.997%, as calculated by 21,143/780,000,000 bp, assumes complete homozygosity of the genome reference donor. Was this a homozygous fish? Otherwise, these SNPs could represent heterozygous loci within this fish or could represent assembly consensus artifact. This is also confounded with potential misalignment of Illumina reads in repetitive regions. Thus, an 'accuracy' estimate is complicated and hard to estimate.

Minor corrections:
Will the RRID citations be replaced with URLs?
Pdf page 7, Line 16 - SDS molecules were quenched. Is "quenched" the correct term?
Pdf page 7, Line 39 - Please provide a reference for the 'previous study'.
Pdf page 8, Line 3 - do you mean 'contig number' instead of 'sequence number'?
Pdf page 8, Line 23 - Which BUSCO database was used for this comparison?
Pdf page9, lines 13 and 31 - 'with a maximal e-value'

Level of Interest

Please indicate how interesting you found the manuscript: Choose an item.

Quality of Written English

Please indicate the quality of language in the manuscript: Choose an item.

Declaration of Competing Interests

Please complete a declaration of competing interests, considering the following questions:

- Have you in the past five years received reimbursements, fees, funding, or salary from an organisation that may in any way gain or lose financially from the publication of this manuscript, either now or in the future?
- Do you hold any stocks or shares in an organisation that may in any way gain or lose financially from the publication of this manuscript, either now or in the future?
- Do you hold or are you currently applying for any patents relating to the content of the manuscript?
- Have you received reimbursements, fees, funding, or salary from an organization that holds or has applied for patents relating to the content of the manuscript?
- Do you have any other financial competing interests?
- Do you have any non-financial competing interests in relation to this paper?

If you can answer no to all of the above, write 'I declare that I have no competing interests' below. If your reply is yes to any, please give details below.

I declare that I have no competing interests.

I agree to the open peer review policy of the journal. I understand that my name will be included on my report to the authors and, if the manuscript is accepted for publication, my named report including any attachments I upload will be posted on the website along with the authors' responses. I agree for my report to be made available under an Open Access Creative Commons CC-BY license (http://creativecommons.org/licenses/by/4.0/). I understand that any comments which I do not wish to be included in my named report can be included as confidential comments to the editors, which will not be published.

I agree to the open peer review policy of the journal

To further support our reviewers, we have joined with Publons, where you can gain additional credit to further highlight your hard work (see: https://publons.com/journal/530/gigascience). On publication of this paper, your review will be automatically added to Publons, you can then choose whether or not to claim your Publons credit. I understand this statement. Yes
