# Supplementary material for: Chromosomal-level assembly of yellow catfish genome using third-generation DNA sequencing and Hi-C analysis
Source: Gigascience. 2018 Sep 20;7(11):giy120. doi: 10.1093/gigascience/giy120 (PMC6228179; doi:10.1093/gigascience/giy120)
Supplement: Reviewer_2_Report_(Original_Submission) -- Christiaan Henkel — 7/29/2018 Reviewed [file giy120_reviewer_2_report_(original_submission).pdf]

Reviewer Report

Title: **Chromosomal-level assembly of yellow catfish genome using third-generation DNA sequencing and Hi-C analysis**

Version: **Original Submission** **Date:** 7/29/2018

Reviewer name: **Christiaan Henkel**

Reviewer Comments to Author:

This manuscript describes the assembly of the yellow catfish genome, using state-of-the-art methodology. I have a few questions/comments on the text, methodology and results, that I would ask the authors to address:

Page 2, line 12: ' genome character evaluation', I assume this refers to nucleotide identity (using Illumina sequence) as contrasted with structural assembly (using PacBio data)? Also, what were the lengths of the Illumina reads?
Page 3, line 2: I am not familiar with the Genome Puzzle Master method. Perhaps you could describe the methodology is some detail. For example, what are its assumptions when merging assemblies? Do these fit two long-read assemblies as input data? How does the method end up with a much larger (730 Mbp) assembly than either of the inputs (both around 690 Mbp)?
Page 3, line 5: plion -> Pilon
Page 3, HiC description: This section is much more detailed than the others, perhaps streamline this a bit. In the methods, I actually miss the crosslinking step?
Page 3, lines 40/55: please cite the ' previous study'/'previous reports'
Page 4, line 33: 'homologous SNP' -> homozygous SNP?
Figure 2: Please add a scale for the heatmap. Also, the assembly size used appears to be a 690 Mbp one instead of the final 730 Mbp assembly?
Figure 3: I a quite sure there are more than five teleost assemblies with contig N50s over 100 kbp. The scaffold N50 scale is, for the interesting assemblies, less of a measure of assembly quality than an illustration of chromosome length.

General comment: as a major biological interest for the use of this genome assembly is the study of sex determination, and you used a female (XY) specimen, I assume you could already identify the two sex chromosomes in the assembly (using either coverage or heteryzygosity)?

Level of Interest

Please indicate how interesting you found the manuscript: Choose an item.

Quality of Written English

Please indicate the quality of language in the manuscript: Choose an item.

Declaration of Competing Interests

Please complete a declaration of competing interests, considering the following questions:

- Have you in the past five years received reimbursements, fees, funding, or salary from an organisation that may in any way gain or lose financially from the publication of this manuscript, either now or in the future?
- Do you hold any stocks or shares in an organisation that may in any way gain or lose financially from the publication of this manuscript, either now or in the future?
- Do you hold or are you currently applying for any patents relating to the content of the manuscript?
- Have you received reimbursements, fees, funding, or salary from an organization that holds or has applied for patents relating to the content of the manuscript?
- Do you have any other financial competing interests?
- Do you have any non-financial competing interests in relation to this paper?

If you can answer no to all of the above, write 'I declare that I have no competing interests' below. If your reply is yes to any, please give details below.

I declare that I have no competing interests.

I agree to the open peer review policy of the journal. I understand that my name will be included on my report to the authors and, if the manuscript is accepted for publication, my named report including any attachments I upload will be posted on the website along with the authors' responses. I agree for my report to be made available under an Open Access Creative Commons CC-BY license (http://creativecommons.org/licenses/by/4.0/). I understand that any comments which I do not wish to be included in my named report can be included as confidential comments to the editors, which will not be published.

I agree to the open peer review policy of the journal

To further support our reviewers, we have joined with Publons, where you can gain additional credit to further highlight your hard work (see: https://publons.com/journal/530/gigascience). On publication of this paper, your review will be automatically added to Publons, you can then choose whether or not to claim your Publons credit. I understand this statement. Yes
