# Supplementary material for: Chromosomal-level assembly of yellow catfish genome using third-generation DNA sequencing and Hi-C analysis
Source: Gigascience. 2018 Sep 20;7(11):giy120. doi: 10.1093/gigascience/giy120 (PMC6228179; doi:10.1093/gigascience/giy120)
Supplement: Reviewer_3_Report_(Original_Submission) -- Paolo Franchini — 8/2/2018 Reviewed [file giy120_reviewer_3_report_(original_submission).pdf]

Reviewer Report

Title: Chromosomal-level assembly of yellow catfish genome using third-generation DNA sequencing and Hi-C analysis

Version: Original Submission **Date:** 8/2/2018

Reviewer name: Paolo Franchini

Reviewer Comments to Author:

In this manuscript Gong and colleagues reports the genome assembly of the yellow catfish (Pelteobagrus fulvidraco), an economically important freshwater fish manly farmed in China. This fish species exhibits a remarkable sex dimorphism on growth rate. Considering its economic relevance, the draft genome of the yellow catfish will be a valuable resource to facilitate future research aimed at improving relevant traits, and more generally at addressing ecological and evolutionary questions.

The authors used an adequate amount of sequence data coming from three different technologies (short reads, long reads and Hi-C), and this allowed to generate a robust chromosome-level genome. The workflow to assemble the genome sounds good and it is generally well described, even though some steps need to be better explained. Further, the authors annotated the genome using a combination of ab initio and homology-based methods that allowed them to identify a number of genes that is comparable to what has usually been found in other teleost fishes. Finally, they carried out some comparative genomics analyses including a bunch of other fish species in order to place the yellow catfish in well-defined phylogenetic context and to analyse the expansion/contraction of gene families in this lineage.
That said, I think that this manuscript needs some revision before to be considered for publication in GigaScience. My main concern is about the language as the manuscript suffers from lack of clarity in several sections. Many sentences would benefit from being re-written and in general all the manuscript should be proofread before to be resubmitted to this or to any other journal.

Minor points:

Abstract, Finding:
- Change "The sequencing results were assembled…" to "The sequencing data were assembled…"

Introduction:
- The introduction is quite short. I would suggest the authors to expand the first section focusing on the species description, what are its distinctive traits, what type of studies have been done so far and to address which questions etc.

- As it appears here for the first time, please introduce "Hi-C" with its full name "Chromosome conformation capture"

Sample and sequencing:
- "…platform according to the according…"

- PacBio flow cell should be "PacBio SMRT Cell"

Genome quality evaluatuion:
- Which version of BUSCO did you use? And which database? The CVG (Core Vertebrate Genes) or the whole

vertebrate gene set? Please include the number of genes that matched the database used, not only the percentage.

- "Using the Illumina short…". Can the authors explain what they exactly did here?

Conclusion:
- Here at the beginning of the paragraph, I would mention that you also used Illumina short reads.

Software version is missing several times along the text, and different styles are used to cite a software. Please revise and make it consistent.

Table 1:
- I would slightly change the first 2 columns of table 1:
Library type: "short reads", "long reads", "Hi-C"
Sequencing platform: "HiSeq Xten", "PacBio Sequel", "HiSeq Xten",

Figure 1 is quite ugly. I suggest the authors to look for a better image or take a picture themselves.

Figure 3: The legend is not so informative. What are the black and the red dots? I know they indicate different fish species, but what are the criteria to make them red or black? Please add this info in the legend.

Level of Interest

Please indicate how interesting you found the manuscript: Choose an item.

Quality of Written English

Please indicate the quality of language in the manuscript: Choose an item.

Declaration of Competing Interests

Please complete a declaration of competing interests, considering the following questions:

- Have you in the past five years received reimbursements, fees, funding, or salary from an organisation that may in any way gain or lose financially from the publication of this manuscript, either now or in the future?
- Do you hold any stocks or shares in an organisation that may in any way gain or lose financially from the publication of this manuscript, either now or in the future?
- Do you hold or are you currently applying for any patents relating to the content of the manuscript?
- Have you received reimbursements, fees, funding, or salary from an organization that holds or has applied for patents relating to the content of the manuscript?
- Do you have any other financial competing interests?
- Do you have any non-financial competing interests in relation to this paper?

If you can answer no to all of the above, write 'I declare that I have no competing interests' below. If your reply is yes to any, please give details below.

I declare that I have no competing interests.

I agree to the open peer review policy of the journal. I understand that my name will be included on my report to the authors and, if the manuscript is accepted for publication, my named report including any attachments I upload will be posted on the website along with the authors' responses. I agree for my report to be made available under an Open Access Creative Commons CC-BY license (http://creativecommons.org/licenses/by/4.0/). I understand that any comments which I do not wish to be included in my named report can be included as confidential comments to the editors, which will not be published.

I agree to the open peer review policy of the journal

To further support our reviewers, we have joined with Publons, where you can gain additional credit to further highlight your hard work (see: https://publons.com/journal/530/gigascience). On publication of this paper, your review will be automatically added to Publons, you can then choose whether or not to claim your Publons credit. I understand this statement. Yes
