# Supplementary material for: Chromosomal-level assembly of yellow catfish genome using third-generation DNA sequencing and Hi-C analysis
Source: Gigascience. 2018 Sep 20;7(11):giy120. doi: 10.1093/gigascience/giy120 (PMC6228179; doi:10.1093/gigascience/giy120)
Supplement: Response_To_Reviewer_Comments_(Original_Submission).pdf [file giy120_response_to_reviewer_comments_(original_submission).pdf]

Author's Response To Reviewer Comments

Close

Dear Editors:

We are submitting the revised manuscript entitled "Chromosomal-level assembly of yellow catfish genome using third-generation DNA sequencing and Hi-C analysis". You can find our detailed answers to points raised by reviewers in our response letter. We have revised our manuscript according to the comments from the reviewers.

You and other reviewers are high appreciated for your constructive suggestions. We believe the quality and scientific significance of the manuscript has improved greatly due to the feedback of the reviewers. We hope that the paper is now in a form suitable for publication in Giga Science as a Data Note.

Yours sincerely,

Prof. Dr. Jie Mei
College of Fisheries
Huazhong Agricultural University, Wuhan, China
Email: jmei@mail.hzau.edu.cn
Aug 22, 2018

Reviewer reports:
Reviewer #1: This manuscript describes the sequencing, assembly, and analysis of the genome of yellow catfish (Pelteobagrus fulvidraco). The authors utilized long fragment sequencing and Hi-C scaffolding to produce chromosome-level scaffolds, then short sequences to help validate long sequences and correct consensus errors. They provided analyses to estimate gene content. This genome will be useful for basic biological studies of the yellow catfish and also for use in agriculture. The comments below are intended to better clarify the information provided.

Estimated genome size was calculated using kmer-based calculation. This can be performed on unfiltered Illumina data, and kmers with low frequency are removed from the calculation. In their figure, this occurs at a frequency of about 15 - anything below that frequency is untrusted and is likely sequencing artifact. The authors should also make this calculation using other kmer lengths (at least 21-mers and 25-mers) to ensure this is a robust estimate.
Reply: Thanks a lot for the suggestion. We have used the method mentioned by the reviewer and re-analyzed the Illumina data with Kmer of 17, 21, 25 and 27. The estimated genome size ranged from 706 to 714 Mb. We have added the results into the revised manuscript in line 135 and Supplementary Table 1.

The assembly was polished using arrow and pilon (misspelled in line 7). The latest recommendation from the National Human Genome Research Institute is to use pilon to correct only indels because the short Illumina reads can be misaligned within repetitive regions and incorrectly polish the sequence.
Reply：We thanks a lot for the reviewer's reminding. The typo of pilon was correct in the line 156. We have tested the effects of the pilon on the base error correction. The results confirmed that pilon could significantly reduce both substitution and InDel errors (data will

be published in our following paper). Therefore, we applied pilon to correct both snp and InDel for the assembled genome in this work.

Was blood from the genome reference fish used for Hi-C analysis, or was this blood from a different animal?
Reply: We used the same individual for genome reference sequencing and Hi-C experiment. We have added the description in line 164 of the revised manuscript.

There is no information on the average contig length or range of lengths, or the number and distribution of gaps within each chromosomal scaffold. Although the contig N50 is 1.1 Mb, there are still 2,440 contigs in the assembly, which suggests there are many small contigs.
Reply: The reviewer's concern is very important. We have added a length distribution figure (Supplementary Figure 2) in the revised manuscript, showing the range of contig length. We also added a figure (Supplementary Figure 3) to show the length distribution of anchored and unanchored contigs, showing that the length of the unanchored contigs were obviously smaller than the anchored contigs. We therefore speculated that short lengths of unanchored contigs limited effective Hi-C reads mapping, leading to insufficient supporting evidence for their clustering, ordering and orientation on chromosomes. The gap distribution along chromosome was also shown in the Supplementary Figure 4. We found that gaps were enriched on two ends of chromosomes. Gap distribution of on chromosomes could be explained the distribution of repeat at chromosome terminals.

What is the final statistic on contig numbers, length, scaffolds, etc. for the submission?
Reply: We have added the Supplementary Table 2 in the revised manuscript according to the reviewer's suggestion.

How many contigs are there per chromosome? A simple table would suffice.
Reply: The supplementary table 2 were added in the revised manuscript to show the detailed contig information for chromosomes.

What was the average length of the 1,224 contigs that were removed during chromosomal scaffolding?
Reply：Using Hi-C data, we anchored 2,440 of 3,652 contigs into chromosomes. Those contigs (sequence number 1,212) were not removed, but was left in the final assembly as unanchored sequences. The average length of those 1,212 was 35.0 kb, which was significantly lower than that of the whole genome contigs (Supplementary Figure 3).

In Figure 4, the authors place seahorse phylogeny somewhere within teleost phylogeny. They should carefully examine their tree and compare it to previously published phylogenetic trees, with justifications when their results differ from the vast array of available phylogenies.
Reply：The reviewer's concern is very important. We have examined the phylogenetic results with the seahorse genome literature1, and found our result was consistent with the study. We thank the reviewer for the important reminding. We have added the reference in our revised manuscript in line 281.

There are no Figure Legends, and the information on the figures is insufficient. Figures should stand alone. For example, in Fig 2, what does the scale of 2-12 represent on the right? In Fig 3, which genomes are included in the black dots? In Supp Fig2, what do the

colors represent?
Reply：We thank the reviewer for the reminding. We have added the detailed legends for figures. The color bar in Figure 2 illuminated the logarithm of the contact density from red (high) to white (low) in the plot. The statistics of 44 teleost genomes (43 public and the P. fulvidraco genome) were included in the Fig 3. We have added Supplementary Table 3 to include the statistics of genomes in Figure 3. In Supp Fig 2, the color represented the value of density. We have added the detailed legends for figures and tables in the revised manuscript.

Supplementary Figure 3 provides useful information and demonstrates the quality of the assembly. If Figures are limited, the authors may consider exchanging this with Figure 3.
Reply： We thank the reviewer's constructive suggestion. We have added the Supplementary Figure 3 as Figure 4 in the revised manuscript.

Pdf page 8, Line 32 -The accuracy of 99.997%, as calculated by 21,143/780,000,000 bp, assumes complete homozygosity of the genome reference donor. Was this a homozygous fish? Otherwise, these SNPs could represent heterozygous loci within this fish or could represent assembly consensus artifact. This is also confounded with potential misalignment of Illumina reads in repetitive regions. Thus, an 'accuracy' estimate is complicated and hard to estimate.
Reply：The reviewer is correct that the "accuracy" of the genome assembly was complicated and hard to estimated. To avoid the mis-understanding, we have deleted the sentence of the genome accuracy from our manuscript.

Minor corrections:
Will the RRID citations be replaced with URLs?
Reply：We used the RRID for software used in this work because the GigaScience journal recommends the RRID. We have added a list of software and URLs at the end of the revised manuscript.

Pdf page 7, Line 16 - SDS molecules were quenched. Is "quenched" the correct term?
Reply: Thank for the reviewer's reminding. In this experimental step, Triton X-100 was used to quench the SDS to prevent it from denaturing enzymes in subsequent steps. We used the similar experimental steps and description as the following the reference2.

Pdf page 7, Line 39 - Please provide a reference for the 'previous study'.
Reply: Thanks for the reminding. We have added the citation in the revised manuscript (line 183).

Pdf page 8, Line 3 - do you mean 'contig number' instead of 'sequence number'?
Reply: We have corrected the sequencing number to contig number as reviewer's suggestion (line 204).

Pdf page 8, Line 23 - Which BUSCO database was used for this comparison?
Reply: The actinopterygii_odb9 database in BUSCO was used in our analysis. We have added the information in our revised manuscript in line 225.

Pdf page9, lines 13 and 31 - 'with a maximal e-value'
Reply：We have corrected the manuscript according to the reviewer's suggestion in line 258

and 268.

Reviewer #2: This manuscript describes the assembly of the yellow catfish genome, using state-of-the-art methodology. I have a few questions/comments on the text, methodology and results, that I would ask the authors to address:

Page 2, line 12: ' genome character evaluation', I assume this refers to nucleotide identity (using Illumina sequence) as contrasted with structural assembly (using PacBio data)? Also, what were the lengths of the Illumina reads?
Reply：Sorry for the confound to the reviewer. Here "genome character evaluation" means using NGS data to evaluated the genome size, the level of heterozygosity and repeat content in the genome. We have added the description to clarify in line 114-115. The length of Illumina reads was 150 bp.

Page 3, line 2: I am not familiar with the Genome Puzzle Master method. Perhaps you could describe the methodology is some detail. For example, what are its assumptions when merging assemblies? Do these fit two long-read assemblies as input data? How does the method end up with a much larger (730 Mbp) assembly than either of the inputs (both around 690 Mbp)?
Reply：Genome puzzle master (GPM) is a tool to build and edit pseudomolecules from fragmented sequences using sequence relationships.3 Since overlap information among contigs from two genomes can be used to guide the genome assembly, one important application of the GPM is to improve the genome assembly through sequence-to-sequence alignments. Based on complements of two genomes, the contig could be elongated and the gaps are filled by sequences bridging two contigs. The method was used to improved the rice genome assemblies based on PacBio sequencing.4 We have added the more information and the reference for the application of GMP in line 146-151 of the revised manuscript.

Page 3, line 5: plion -> Pilon
Reply: Thanks for the reviewer for the reminding. We have correct the typo in line 156 of the revised manuscript.

Page 3, HiC description: This section is much more detailed than the others, perhaps streamline this a bit. In the methods, I actually miss the crosslinking step?
Reply：Thanks a lot for the reviewer. We have added the detailed steps for the crosslinking step for our Hi-C experiments in line 166-170 of the revised manuscript.

Page 3, lines 40/55: please cite the ' previous study'/'previous reports'
Reply：We have added the citation for the previous study to support the sentence in line 183.

Page 4, line 33: 'homologous SNP' -> homozygous SNP?
Reply: Thanks a lot for the reviewer. We have corrected the typo in the line 235 of the revised manuscript.

Figure 2: Please add a scale for the heatmap. Also, the assembly size used appears to be a 690 Mbp one instead of the final 730 Mbp assembly?
Reply：Thanks for the reviewer's reminding. We only illuminate the contact heatmap for

sequences anchored in chromosomes, with a total length of 690 Mb. We have added the information in the legend of the figure.

Figure 3: I a quite sure there are more than five teleost assemblies with contig N50s over 100 kbp. The scaffold N50 scale is, for the interesting assemblies, less of a measure of assembly quality than an illustration of chromosome length.
Reply：We have included a supplementary table 3 for the fish species that we used in Figure 3. However, not every fish were assembled into chromosome level. Therefore, we only showed scaffold N50 in the Y axis.

General comment: as a major biological interest for the use of this genome assembly is the study of sex determination, and you used a female (XY) specimen, I assume you could already identify the two sex chromosomes in the assembly (using either coverage or heteryzygosity)?
Reply: We used XX female for genome assembly. The reviewer is correct. We have developed sex-specific markers in our previous studies5 and could help us to identified putative sex chromosome. The application of the genome on the sex-determination studies of the yellow catfish will be illuminated in our following reports.

Reviewer #3: In this manuscript Gong and colleagues reports the genome assembly of the yellow catfish (Pelteobagrus fulvidraco), an economically important freshwater fish manly farmed in China. This fish species exhibits a remarkable sex dimorphism on growth rate. Considering its economic relevance, the draft genome of the yellow catfish will be a valuable resource to facilitate future research aimed at improving relevant traits, and more generally at addressing ecological and evolutionary questions.

The authors used an adequate amount of sequence data coming from three different technologies (short reads, long reads and Hi-C), and this allowed to generate a robust chromosome-level genome. The workflow to assemble the genome sounds good and it is generally well described, even though some steps need to be better explained. Further, the authors annotated the genome using a combination of ab initio and homology-based methods that allowed them to identify a number of genes that is comparable to what has usually been found in other teleost fishes. Finally, they carried out some comparative genomics analyses including a bunch of other fish species in order to place the yellow catfish in well-defined phylogenetic context and to analyse the expansion/contraction of gene families in this lineage.

That said, I think that this manuscript needs some revision before to be considered for publication in GigaScience. My main concern is about the language as the manuscript suffers from lack of clarity in several sections. Many sentences would benefit from being re-written and in general all the manuscript should be proofread before to be resubmitted to this or to any other journal.

Minor points:
Abstract, Finding:
- Change "The sequencing results were assembled…" to "The sequencing data were assembled…"
Reply: Thanks a lot for the reviewer's correction. We have revised the manuscript in line 39.

Introduction:
- The introduction is quite short. I would suggest the authors to expand the first section focusing on the species description, what are its distinctive traits, what type of studies have been done so far and to address which questions etc.
Reply: Thank a lot for the reviewer's constructive suggestion. We have expanded our introduction section for the description and recent research progresses of the yellow catfish in line 67-86 of the revised manuscript.

- As it appears here for the first time, please introduce "Hi-C" with its full name "Chromosome conformation capture"
Reply: Thanks for the reviewer's suggestion. We have added the introduction of Hi-C in the revised manuscript in line 160-165.

Sample and sequencing:
- "…platform according to the according…"
- PacBio flow cell should be "PacBio SMRT Cell"
Reply：We have revised the sentences in line 138 of the revised manuscript.

Genome quality evaluatuion:
- Which version of BUSCO did you use? And which database? The CVG (Core Vertebrate Genes) or the whole vertebrate gene set? Please include the number of genes that matched the database used, not only the percentage.
Reply：BUSCO v3.0 and actinopterygii_odb9 database was used for the genome evaluation. We have added the BUSCO version, database type and detailed output of the analysis in our revised manuscript (line 224-225).

- "Using the Illumina short…". Can the authors explain what they exactly did here?
Reply：The Illumina short reads were aligned to the reference genome, and the SNP loci were called through GATK pipeline. We have added the detailed method in the revised manuscript (line 233-234).

Conclusion:
- Here at the beginning of the paragraph, I would mention that you also used Illumina short reads.
Reply：Thanks for the reminding. We have added the Illumina short read in the revised manuscript in line 304.

Software version is missing several times along the text, and different styles are used to cite a software. Please revise and make it consistent.
Reply：We have added the software version information and revised the citation for the software.

Table 1:
- I would slightly change the first 2 columns of table 1:
Library type: "short reads", "long reads", "Hi-C"
Reply：We have revised the table information according to the reviewer's suggestion in Table 1.

Figure 1 is quite ugly. I suggest the authors to look for a better image or take a picture

themselves.
Reply: We have replaced the Figure 1 in our revised manuscript. Thank the reviewer for the suggestion.

Figure 3: The legend is not so informative. What are the black and the red dots? I know they indicate different fish species, but what are the criteria to make them red or black? Please add this info in the legend.
Reply: The red dots in Figure 3 represented the teleost genomes that totally assembled using long reads from the third-generation sequencing platform. We have added the detailed the legends for all figures and tables in the revised manuscript.

References
1 Qiang, L. et al. The seahorse genome and the evolution of its specialized morphology. Nature 540, 395-399 (2016).
2 Belton, J. M. et al. Hi-C: a comprehensive technique to capture the conformation of genomes. Methods 58, 268-276 (2012).
3 Zhang, J. et al. in International Plant and Animal Genome Conference Xx.
4 Zhang, J. et al. Extensive sequence divergence between the reference genomes of two elite indica rice varieties Zhenshan 97 and Minghui 63. Proc Natl Acad Sci U S A 113, E5163 (2016).
5 Wang, D., Mao, H. L., Chen, H. X., Liu, H. Q. & Gui, J. F. Isolation of Y- and X-linked SCAR markers in yellow catfish and application in the production of all-male populations. Animal Genetics 40, 978-981 (2010).

Close
